# Inherent constraints on a polyfunctional tissue lead to a reproduction-immunity tradeoff

**DOI:** 10.1186/s12915-022-01328-w

**Published:** 2022-06-02

**Authors:** Vanika Gupta, Ashley M. Frank, Nick Matolka, Brian P. Lazzaro

**Affiliations:** 1 Department of Entomology, Cornell University, Ithaca, NY USA; 2 Cornell Institute of Host-Microbe Interactions and Disease, Cornell University, Ithaca, NY USA

**Keywords:** Tradeoffs, *Drosophila*, Fat body, Translation, Reproduction, Infection, Single-cell sequencing, Single-nucleus sequencing

## Abstract

**Background:**

Single tissues can have multiple functions, which can result in constraints, impaired function, and tradeoffs. The insect fat body performs remarkably diverse functions including metabolic control, reproductive provisioning, and systemic immune responses. How polyfunctional tissues simultaneously execute multiple distinct physiological functions is generally unknown. Immunity and reproduction are observed to trade off in many organisms but the mechanistic basis for this tradeoff is also typically not known. Here we investigate constraints and trade-offs in the polyfunctional insect fat body.

**Results:**

Using single-nucleus sequencing, we determined that the *Drosophila melanogaster* fat body executes diverse basal functions with heterogenous cellular subpopulations. The size and identity of these subpopulations are remarkably stable between virgin and mated flies, as well as before and after infection. However, as an emergency function, the immune response engages the entire tissue and all cellular subpopulations produce induce expression of defense genes. We found that reproductively active females who were given bacterial infection exhibited signatures of ER stress and impaired capacity to synthesize new protein in response to infection, including decreased capacity to produce antimicrobial peptides. Transient provision of a reversible translation inhibitor to mated females prior to infection rescued general protein synthesis, specific production of antimicrobial peptides, and survival of infection.

**Conclusions:**

The commonly observed tradeoff between reproduction and immunity appears to be driven, in *D. melanogaster*, by a failure of the fat body to be able to handle simultaneous protein translation demands of reproductive provisioning and immune defense. We suggest that inherent cellular limitations in tissues that perform multiple functions may provide a general explanation for the wide prevalence of physiological and evolutionary tradeoffs.

**Supplementary Information:**

The online version contains supplementary material available at 10.1186/s12915-022-01328-w.

## Background

The need to balance multiple physiologically demanding and resource-intensive processes limits the ability of an organism to maximize performance in any one area. When two or more processes depend on a single tissue or resource pool, they unavoidably constrain each other, resulting in tradeoffs between the associated traits. Such tradeoffs are central to life history theory and affect the health, fitness, and evolution of all living organisms [1–3]. Reproduction and immunity are two traits that trade off with each other across a broad diversity of systems [4, 5] but the mechanisms and physiological constraints that underlie this tradeoff are poorly understood. In *Drosophila melanogaster* females, mating results in a rapid, endocrinologically-mediated drop in resistance to bacterial infection [6]. We hypothesized that this tradeoff arises due to physiological constraints of using the same tissue, the abdominal fat body, for both reproductive investment and systemic immunity, and that understanding the basis for this tradeoff could serve as a model for understanding constraints on polyfunctional tissues in general.

The insect fat body is a highly multifunctional tissue that is engaged in central metabolic regulation, nutrient storage, detoxification of xenobiotics, reproductive egg provisioning, and mounting of systemic immune responses [7]. Thus, this single tissue performs the functions of several vertebrate organs. The fat body is remarkably dynamic. For example, a bacterial infection significantly changes the expression of several hundred genes in the fat body of *Drosophila melanogaster*, including as much as 1000-fold induction of genes encoding antimicrobial peptides and marked down-regulation of glycolytic and basal metabolic pathways [8–10]. Upon mating and sperm storage, the same tissue significantly upregulates genes involved in egg provisioning as the females increase their investment egg production [7]. Reproduction and immune responses are both energetically demanding [11] and a female may need to simultaneously execute these processes as well as others. Given the finite number of cells and limited capacity for transcription and translation within each cell, how does one tissue achieve so many functions at once? Is the tissue composed of specialized subpopulations of cells that are individually devoted to each function? Or do all cells of the tissue perform all functions to a limited degree? When the tissue responds to stimulus, do the identities or sizes of cellular subpopulations change, or does each cell of the tissue alter its transcriptional profile in concert? Does the simultaneous execution of multiple processes by the single tissue constrain immune performance?

## Results

**Fig. 1:**
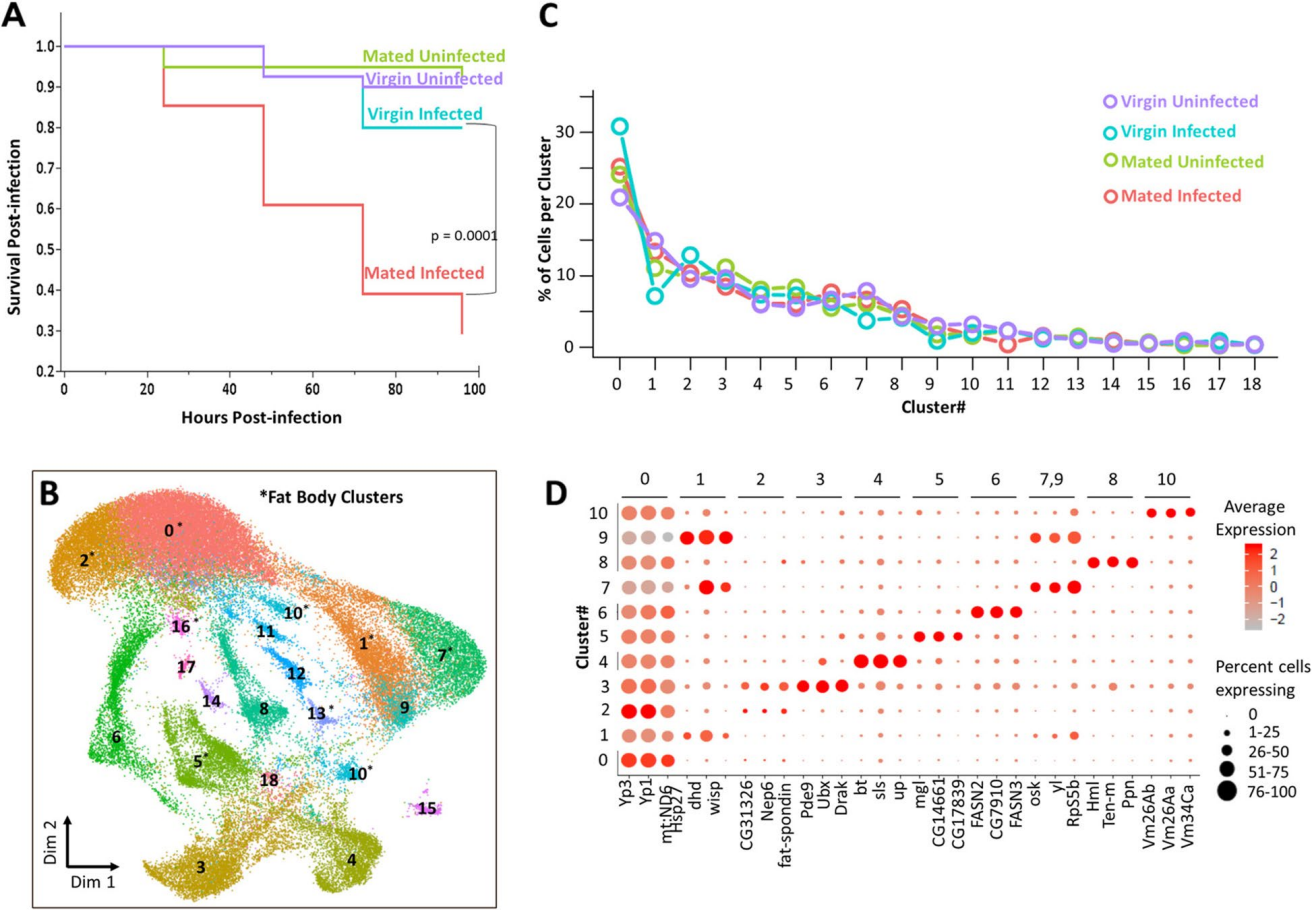
Single-nucleus sequencing of *Drosophila* fat body tissue. **A** Cox proportional hazard analysis showed that the mated *Drosophila melanogaster* females have significantly lower survival than virgin females (*n* = 40; *p* = 0.0001) after infection with the Gram-negative bacterium *Providencia rettgeri*. Survival of uninfected virgin and mated females was not different over four days. **B** Combined Uniform Manifold Approximation and Projection (UMAP) of 56,000 nuclei from two replicates each of Virgin-Uninfected, Virgin-Infected, Mated-Uninfected, and Mated-Infected colored by their treatment identity. Clusters 0, 1, 2, 5, 7, 10, 11, 12, 13, and 16 marked with an asterisk (*) represent subpopulations of the fat body tissue. **C** Percentage distribution of nuclei from four treatments (*V*irgin-*U*ninfected, *V*irgin-*I*nfected, *M*ated-*U*ninfected, and *M*ated-*I*nfected) across 19 clusters. All clusters are present in constant proportion across all four treatments. **D** Dot Plot showing expression of marker genes per cluster for the eleven largest clusters. Average scaled expression of a marker gene across all cells is represented by the color gradient and dot size represents the percentage of cells that express the marker in each cluster. The cluster labels above the figure indicate the three genes (*x*-axis) that are most strongly associated with that cluster. Genes *hsp27* and *wisp* are also top markers for Cluster 9 (see Additional file 1 for more information on cluster properties and markers)

To begin address these questions, we performed single-nucleus RNA sequencing (snRNAseq) on the fat bodies of *D. melanogaster* females in a replicated factorial design combining mating and bacterial infection. Mature adult female *D. melanogaster* were either mated in order to activate reproductive investment (M_) or held as virgin to limit reproductive investment (V_) and, 24 h later, were either given a systemic bacterial infection with *Providencia rettgeri* to stimulate an immune response (_I) or were held uninfected (_U). We observed significantly lower survivorship of Mated-Infected (MI) females than Virgin-Infected (VI) females over 3 days post-infection (*p* = 0.0001; Fig. 1A) in accordance with previous observations [12, 13] and demonstrating the expected tradeoff. We repeated each factorial treatment (VU, VI, MU, MI) in two independent biological replicates to generate a total of 8 samples for snRNAseq. From each sample, we dissected and pooled fat bodies from the abdomens of 40 female flies at 6 h after the infection treatment (approximately 30 h after mating for the mated females). The gut and ovaries are easily removed from the fat body tissue, but other cell types such as oenocytes, muscle cells, and hemocytes are harder to separate from fat body tissues and thus were co-isolated. We purified individual nuclei from the pooled tissues using a Dounce homogenizer followed by centrifugation onto a sucrose cushion [14]. We performed snRNAseq using the 10X Genomics Chromium platform, loading at least 7000 nuclei per sample and sequencing at least 16,000 reads per nucleus for a minimum of 112 million reads per sample.

We identified 19 expression clusters representing distinct cellular subpopulations (Fig. 1B, Additional file 1: Fig. S1, S2, Table S1) with 90% of the nuclei present in the eleven most abundant clusters (Fig. 1C). We assigned putative functional identities to each cluster based on the significantly high expression (*p*-adj <0.01) of diagnostic marker genes. Expression of top marker genes for the first eleven clusters is shown in Fig. 1D, and a full list of key expressed genes can be found in Additional file 1 in Table S2 and Fig. S3. The Supplementary Online Material contains detailed descriptions of the expression patterns and inferred functions for each cluster. We found significantly high expression of marker genes that are conventionally associated with fat body in six major clusters: 0, 1, 2, 5, 7, and 10. These six clusters contain approximately 60% of all the nuclei sequenced. An additional 5% of nuclei map to low-abundance fat body clusters (Clusters 11, 12, 13, and 16). These data demonstrate that the fat body tissue is composed of heterogeneous cell subtypes. Clusters 0 and 2 were defined by high expression of *yolk proteins 1* and *3* (Fig. 1D), while Clusters 1, 5, 7, and 10, respectively, had high expression of *deadhead*, *megalin*, *oskar*, and *vitelline membrane 26Ab*. All these marker genes are associated with oogenesis and egg development. The relative size of these clusters did not change significantly across the four replicated treatment groups (Fig. 1C), indicating that the fat body does not respond to mating or infection by shifting the proportional representation of these specific cellular subpopulations. Our study has identified multiple additional gene markers that could be used to label fat body subpopulations in addition to previously characterized fat body marker genes *yp3* and *yp1* (Additional file 1: Table S2). There are currently no reporter constructs driven by the promoters for these genes, but eventual construction and validation of those tools will allow future analyses including determination of the spatial structure of cellular subpopulations within the tissue.

We infer that the remaining 35% of nuclei do not come from fat body tissue. Based on previously well-characterized cell-specific transcriptional markers, we determined that Cluster 4 is muscle (7% of sequenced nuclei), Cluster 6 is oenocytes (7%), Cluster 8 is hemocytes (5%), and Cluster 9 is uncharacterized (2%). Cluster 3 (10% of sequenced nuclei) remains uncharacterized but shows properties similar to both fat body and pericardial cells (see detailed description in Supplement, Additional file 1: Fig. S4). These tissues are physically contiguous with the fat body, and interact with and have partially overlapping functions with the fat body tissue [15, 16]. Using an available driver for a gene that marks Cluster 3 (*nARCH7α*>Gal4), we determined microscopically that Cluster 3 cells are not fat body cells despite exhibiting gene expression patterns overlapping with the fat body tissue.

Upon mating, *D. melanogaster* females store sperm and begin to lay fertilized eggs, which requires increased investment in oogenesis [17]. We asked whether the investment in reproduction varied across the six distinct subpopulations of the fat body tissue by cluster-specific differential gene expression analysis. When comparing virgin and mated females (24 h post-mating) in the absence of infection, we found 186 differentially expressed genes across the six clusters with 145 genes significantly upregulated and 41 genes significantly downregulated (FDR <0.01; Additional file 2). We observed that none of the 186 genes were differentially regulated across all the six subpopulations (Additional file 1: Fig. S5A) while 123 (66%) of these genes were differentially regulated in only one of the six subpopulations (Additional file 1: Fig. S5A). For example, egg provisioning genes *yp1* and *yp3* were upregulated (Fig. S6A) across four different clusters (Additional file 1) while *yp2* was upregulated in only one cluster (Additional file 2). This indicates that the response to and investment in mating is heterogenous across fat body subpopulations. GO enrichment analysis of differentially regulated genes in each of the six subpopulations showed enrichment for diverse functions (Additional file 3). Upregulated genes in both Clusters 0 and 1 were enriched for one-carbon metabolism but mediated by two different mechanisms: s-adenosyl methionine (SAM; Cluster 0) and folate (Cluster 1). Cluster 1 also showed enriched upregulation of genes encoding ribosomal proteins, which were downregulated in Cluster 2. Upregulated genes in Cluster 2 showed enrichment for amino acid biosynthesis. We identified metabolic and detoxification pathways enriched in genes upregulated in Cluster 5, and upregulated genes in both Clusters 7 and 10 were related to phospholipase A1 activity. Therefore, while all six fat body subpopulations respond to mating stimulus, their heterogeneous response suggests subfunctionalization of the cellular populations.

The fat body mounts an intense and rapid immune response to bacterial infection [9, 18] so we asked whether the whole tissue is engaged in that response or whether it maps to a restricted set of subpopulations. The answer, interestingly, is both. All clusters showed significant upregulation of immune response genes in both mated and virgin females at 6 h after infection, including genes that encode secreted antimicrobial peptides (Additional file 1: Figs.S6B, S6C). However, the precise expression patterns were heterogeneous after infection, with particular combinations of immune genes induced most strongly in different subsets of clusters. Across the six major fat body subpopulations, 90 genes were induced by infection in both virgin and mated females. However, 157 genes showed significant induction after infection in virgin females compared to 101 genes in mated females (Additional files 4 and 5), indicating a negative impact of mating on the transcriptional response to infection. We found 6.4% genes to be differentially regulated across six fat body subpopulations in virgins (Additional file 4, Additional file 1: Fig. S5B) while 5.7% were differentially regulated across every subpopulation in mated females (Additional file 5, Additional file 1: Fig. S5C). Around 19.7% genes were differentially regulated in 4 or more of the six subpopulations in virgins compared to 14.6% in mated females. We observed that 50.9% of differentially expressed genes in virgins and 53.8% of differentially expressed genes in mated were regulated in only one of the six subpopulations (Additional file 1: Figs.S5B, S5C). To understand the functional heterogeneity of the genes expressed in each cluster, we performed cluster-specific GO enrichment analysis of the genes that are differently expressed after infection in mated and virgin females separately (Additional file 6). Protein processing and secretion was a significantly enriched function of upregulated genes in Clusters 0 and 2 in both virgin and mated flies (Additional file 6). Downregulation of ribosome constituents was observed in Clusters 1 and 2 of mated flies and Cluster 2 of virgin females. Oxidative phosphorylation was downregulated in Clusters 0 and 5 in virgin females and Cluster 5 in mated. Additionally, mated females showed downregulated organic acid metabolism and fatty acid biosynthesis in Clusters 0 and 7, respectively. These data reveal heterogeneity in infection response across the fat body (Figs. S5D, S5E) and demonstrate that the tissue-wide transcriptional response to infection also varies between virgin and mated females.

**Fig. 2:**
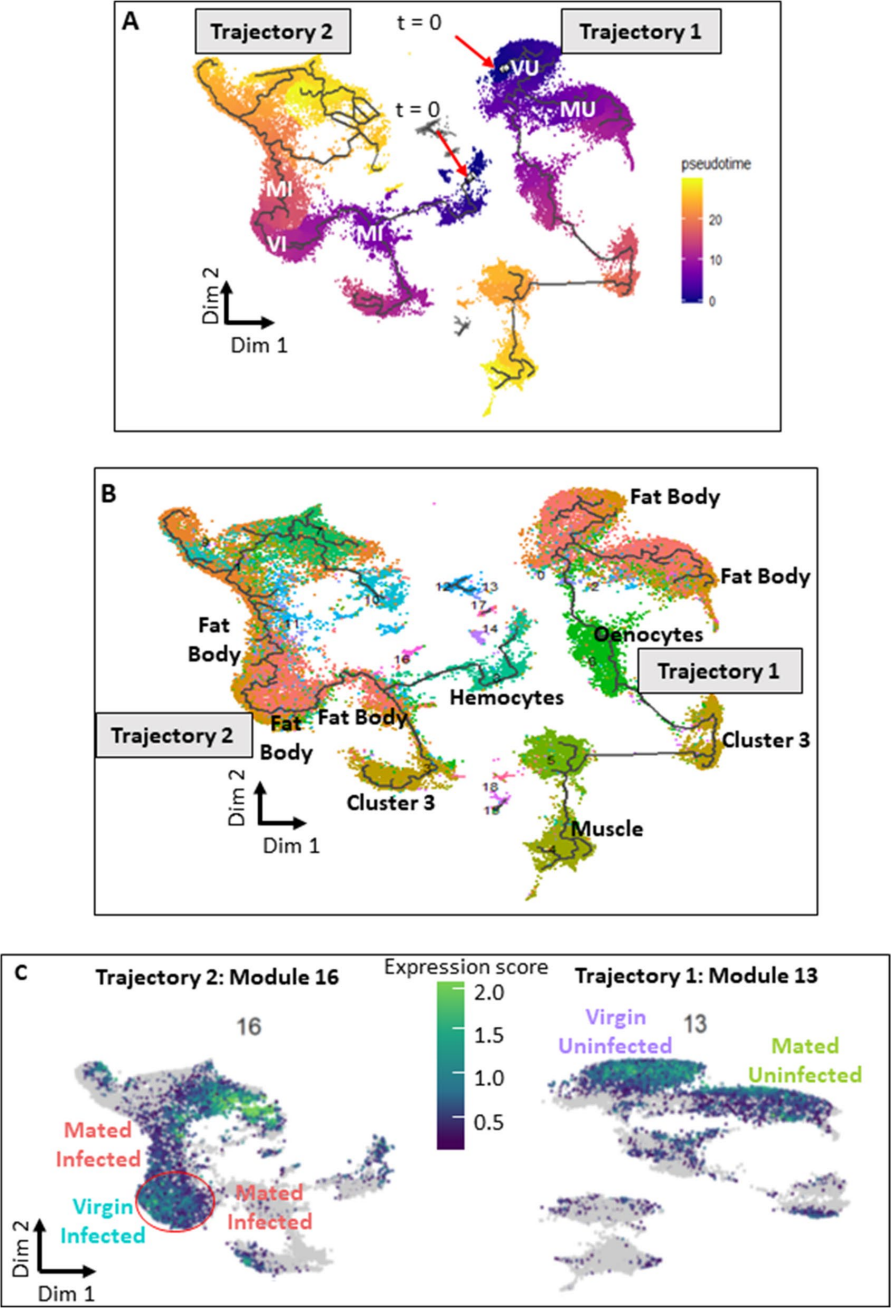
Pseudotime analysis showing differentially expressed gene modules. **A** Pseudotemporal ordering of nuclei along the two trajectories calculated from trajectory-specific (*t* = 0) points. Nuclei from four treatments (*V*irgin-*U*ninfected (VU), *V*irgin-*I*nfected (VI), *M*ated-*U*ninfected (MU), and *M*ated-*I*nfected (MI)) separate at different pseudo-time scales. The trajectories from infected nuclei are completely disjointed from the trajectories of uninfected nuclei, revealing a rapid and dramatic response to infection. **B** Monocle-based trajectory analysis separated nuclei along the two trajectories, colored by their cluster identity (from Fig. 1B), showing that only fat body nuclei are present in both trajectories. Other cell types such as oenocytes and muscle cells are present in Trajectory 1 and hemocytes are present in Trajectory 2, indicating these cell types do not have a strong transcriptional response to infection. **C** UMAP of Module 13 (Trajectory 1) and Module 16 (Trajectory 2) showing low gene aggregate expression scores for Mated-Uninfected (Trajectory 1) and Mated-Infected (Trajectory 2) compared to Virgin-Uninfected and Virgin-Infected, respectively. Gradient of color represents the aggregate expression score with bright color indicating a higher aggregate expression score. Each dot represents a single nucleus. GO term analysis showed enrichment for ribosome biogenesis in the two modules (Additional files 7 and 8, Additional file 1: Fig. S8)

Most of the mating- and infection-induced transcriptional changes were heavily driven by Clusters 0, 1, and 2 (Additional file 1: Table S3), representing ~70% of all the nuclei from the six fat body subpopulations. We hypothesized that the involvement of such a large majority of fat body cells in resource-intensive physiological functions might constrain resource allocation, which could be reflected in coordinated regulation of gene expression networks or modules. To identify these modules, we constructed pseudotime trajectories from all the four treatments with Monocle [19–21], representing the transition of cells between differential functional states in response to mating or infection. Pseudotime analysis orients cells based on their transcriptomic profiles, analogous to representing cellular differentiation along a temporal trajectory. Therefore, higher divergence in cellular subpopulations on pseudotime trajectory suggests larger differences in their transcriptomic profiles. For example (Fig. 2A, B), nuclei at *t* = 0 (in blue) in trajectory 1 represented by fat body from VU are transcriptionally most diverged from muscle in yellow at the end of the trajectory. An initial analysis revealed that the infected and uninfected fat body cells resolved into two completely disjointed trajectories defined by infection status. Trajectory 1 contained a majority of nuclei from VU and MU treatments while Trajectory 2 contained a majority of nuclei from VI and MI (Fig. 2A, Additional file 1: Fig. S7). Analysis of the subset of clusters conclusively identified to be fat body (Fig. S9A) (Clusters 0, 1, 2, 5, 7, and 10) show the same partitioning of nuclei based on infection treatment (Additional file 1: Fig. S9B). This suggests that fat body cells rapidly and dramatically change expression profile upon infection with no intermediate states visible at the 6-h post-infection sampling time point. Only fat body cells (inferred using Seurat-based cluster analysis) were present in both of these trajectories (Fig. 2B). Other co-isolated cell types were present in only one of the two trajectories; indicating that they are not strongly transcriptionally responsive to infection. Using Louvain clustering in the two trajectories, we identified several modules of co-regulated genes that were enriched for specific functional ontologies.

**Fig. 3:**
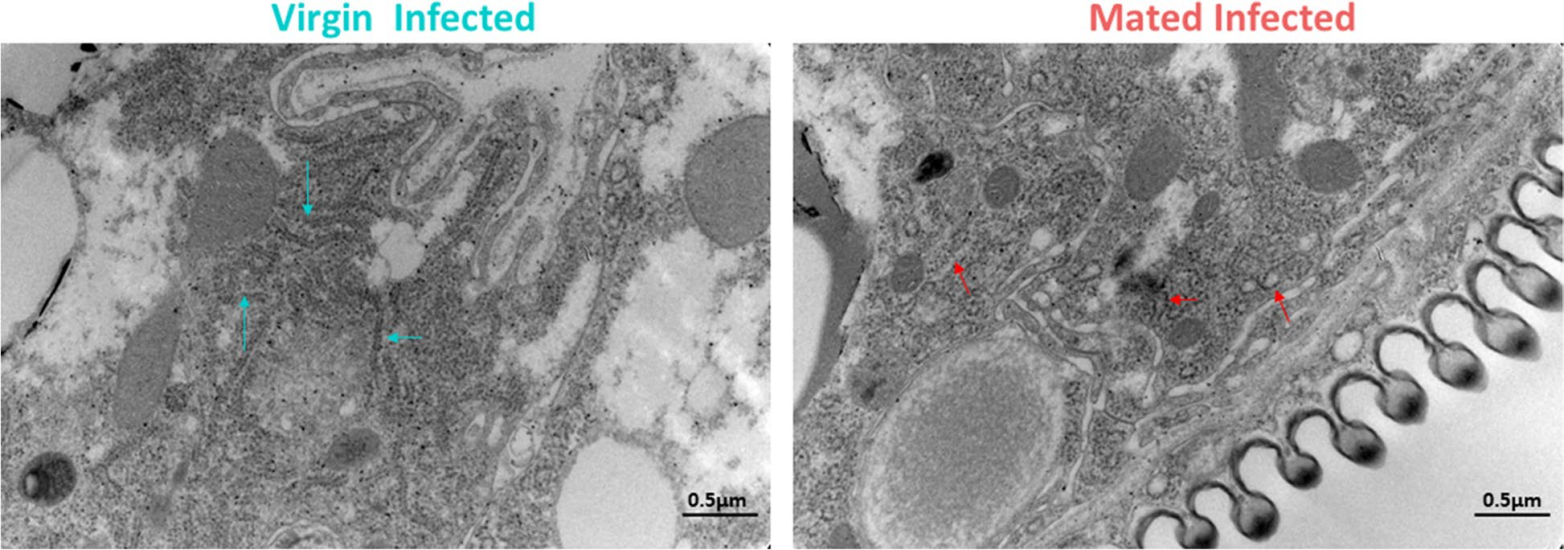
Electron micrographs of endoplasmic reticulum in the fat body (ER). Representative image (*n* = 4–5 images per treatment) showing dilation of ER membrane indicative of ER stress (right panel, red arrows) observed in fat body cells from Mated-Infected females. Blue arrows show constricted ER membranes in Virgin-Infected samples

In Trajectory 1, we identified a module (Module 13, Fig. 2C, Additional file 7) with low aggregate expression score that was enriched in ribosome biogenesis (Additional file 1: Fig. S8) in Mated-Uninfected nuclei (MU) relative to Virgin-Uninfected nuclei (VU), including CAP-dependent translation initiation factors. Surprisingly, the same set of genes with the addition of one gene (*O-fucosyltransferase 2*) had a low aggregate expression score in Mated-Infected (MI) nuclei contrasted to Virgin-Infected nuclei (VI) in Trajectory 2 (Module 16, Fig. 2C, Additional file 8). This pattern was apparent both in the full data and when the analysis was restricted to definitive fat body clusters (Fig. S9C, S9D), and led us to hypothesize that suppressed ribosome biogenesis might cause reduced immunological protein synthesis in females that have been mated and are reproductively active. Furthermore, a subset of MI nuclei showed high expression of a module enriched in protein folding and degradation (Additional file 1: Fig. S10) including genes involved in ER stress and unfolded protein response (UPR; Additional file 9). Electron microscopy confirmed dilated ER membranes in MI fat bodies (Fig. 3), indicative of ER stress [22]. Since alleviation of ER stress is often attained via suppression of ribosome biogenesis to limit protein synthesis in the cell [22, 23], this observation supported our hypothesis that reduced capacity of mated females to produce immune-related proteins could be a key factor underlying the observed reproduction-immunity tradeoff.

**Fig. 4:**
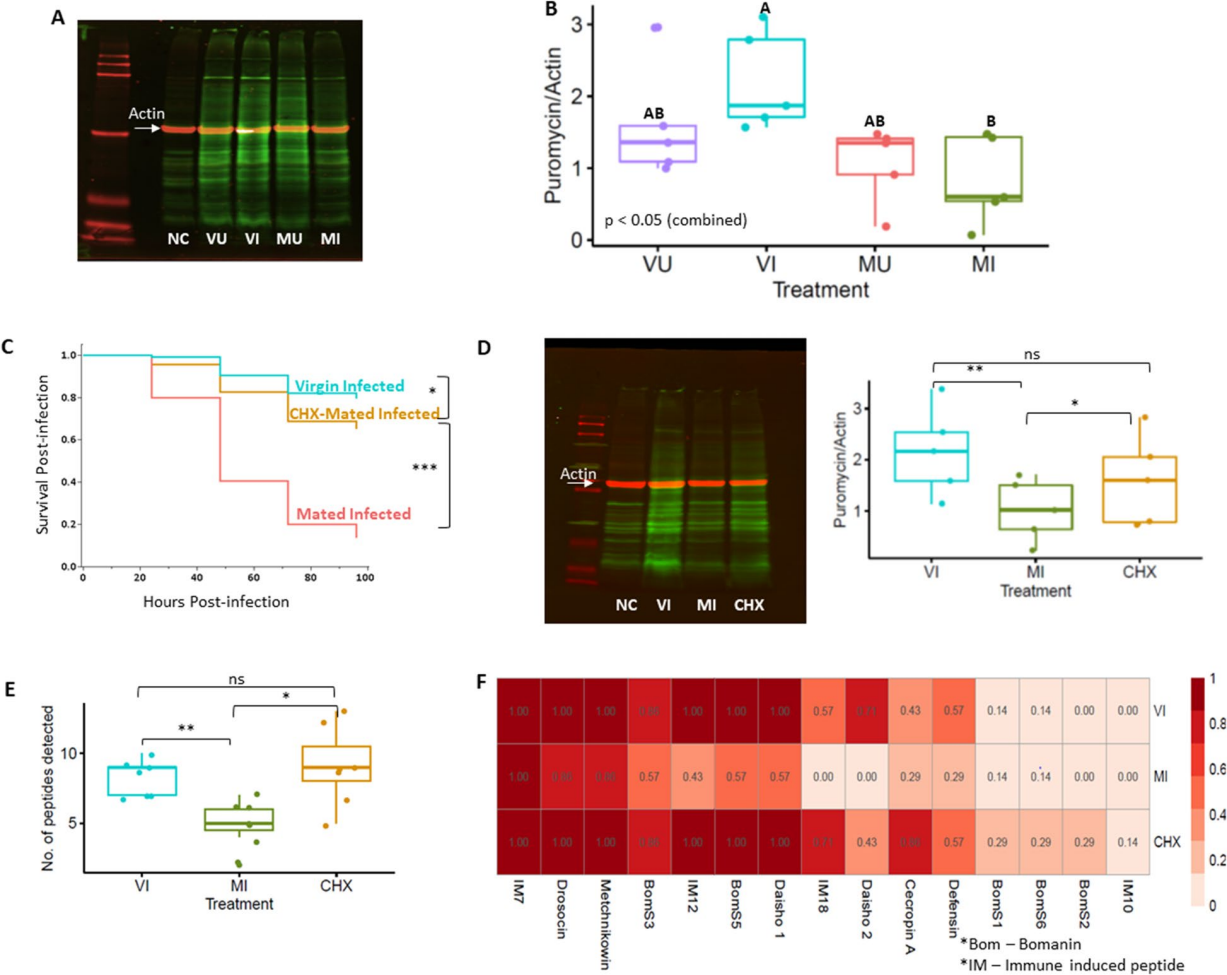
Effect of mating and infection on protein synthesis. **A** Representative image of puromycin incorporation in nascent polypeptides of the fat body tissue detected using anti-puromycin antibody and Western Blotting. Secondary antibodies labeled with different fluorophores detected puromycin (Green, 800nm) and Actin (Red, 700 nm). The fat bodies from Mated-Infected (MI) produce noticeably less protein than those of the other treatments. Virgin-Uninfected (VU), Virgin-Infected (VI), Mated-Uninfected (MU), and Mated-Infected (MI) represent the four treatments. Negative control (NC) (**A** and **D**) shows proteins from fat body tissues which were not incubated with puromycin. **B** Quantification of relative protein synthesis using puromycin incorporation from four treatments (VU, VI, MU, and MI) (*n* = 5, 10 flies per treatment). Treatments not connected by the same letter are significantly different (Tukey’s HSD, *p* < 0.05). Virgin-Infected females synthesize significantly more protein than Mated-Infected females. **C** Cox proportional hazard analysis showing rescued post-infection survival (*p* < 0.0001) of cycloheximide (CHX) pre-treated mated females (CHX- Mated-Infected) compared to non-treated Mated-Infected (*n* = 35–40 flies per treatment per replicate, three independent replicates). **D** Representative Western blot image of puromycin incorporation from CHX pre-treatment of mated females (CHX). CHX treatment partially rescues protein synthesis in response to infection compared to non-treated mated females (MI) (paired Student’s *t*-test, *p* < 0.05). Puromycin incorporation was measured after 6 h of infection. **E** The number of unique immune peptides detected per sample in hemolymph samples collected from VI, MI, and CHX females (*n* = 7, 3 flies per sample). Fewer immune peptides were detected in MI compared to VI (paired Student’s *t*-test, *p* < 0.01) and CHX (paired Student’s *t*-test, *p* < 0.05). (F) Heatmap showing the proportion of samples per treatment in which each peptide was detected. Darker colors in the heatmap indicate a higher proportion of samples in which a given peptide was detected. Significance levels: *ns* not significant, (*) *p*<0.05, (**) *p*<0.01, (***) *p*<0.001

To test our hypothesis that Mated-Infected (MI) females may lack sufficient capacity for translation in support of a full immune response to infection, we measured global protein synthesis in fat body tissues representing each of the four different treatments. We re-generated new female flies from each of the four factorial mating and infection treatments, dissected their fat bodies, and applied puromycin incorporation to label nascent polypeptides. Incorporated puromycin was then quantified on Western blots [20, 21]. We observed significant variability in global synthesis rates across the four treatments (one-way ANOVA, *p*=0.02, Additional file 1: Table S4) with a spike in protein synthesis after infection in virgin females (VI) (mean = 2.2, s.d. = 0.69) that fails to occur in mated (MI) females (mean = 0.8, s.d. = 0.61) (Tukey’s HSD, *p* = 0.0005, Fig. 4A, B). These data are consistent with the reduction in ribosome biogenesis inferred from the hypothesis that the fat bodies of MI females are deficient in translation capacity. As the rapidity of an induced immune response is a critical determinant of infection outcome [22, 23], a quantitative reduction or delay in the translation of immune response proteins such as antimicrobial peptides could contribute to the observed increased risk of death from infection in mated females [24, 25].

Since we observed a reduction in protein synthesis in Mated-Infected (MI) flies compared to all other groups and especially compared to Virgin-Infected (VI) females (Fig. 4B), we hypothesized that the high demand of producing reproduction-related proteins in mated females might reduce capacity to translate new proteins in response to infection and could lead to ER stress when reproductive and immunological translational demands are combined in MI females. We predicted that the reproduction-immunity tradeoff could be alleviated if translational investment in reproductive proteins was reduced. To test this hypothesis, we mated females and then placed them on food containing cycloheximide (CHX) for 18 h. CHX reversibly suppresses the production of proteins in eukaryotes such as *Drosophila* [26]. We subsequently transferred flies to food without CHX for 6 h to allow them to clear the drug, and then gave them bacterial infections. Females that were treated with CHX after mating survived infection significantly better than mated females that were not treated with CHX (*p*<0.0001, Fig. 4C). We hypothesized that pre-treatment with CHX would result in a rescue of capacity for post-infection protein production by mated females. As predicted, we observed higher protein synthesis in mated females pre-treated with CHX compared to non-treated females at 6 h after infection (*t*(4) = 3.63, *p*=0.02, Fig. 4D, Additional file 1: Table S5). Crucially, we determined that mated females that were treated transiently with CHX were virgin-like in their ability to produce antimicrobial peptides in response to infection, whereas MI females that had not been treated with CHX were impaired in antimicrobial peptide synthesis. We used MALDI-TOF MS to detect immune-related peptides (Additional file 1: Fig. S12) including directly antimicrobial peptides (<5 kDa) in the hemolymph of VI, MI, and CHX females at 16 h after infection. Out of the 15 unique immune peptides detected (Fig. 4F), we consistently detected a lower abundance of immune peptides in MI flies (Fig. 4E, F) when compared to VI (paired Student’s *t*-test, *p*=0.009) or CHX (paired Student’s *t*-test, *p*=0.03) flies (Additional file 1: Table S6). There was no significant difference in peptide detection between VI and CHX flies (Additional file 1: Table S6). Additional experiments confirmed that CHX has no direct role in the survival of infection (Additional file 1: Fig. S11, see Methods for experimental details). Therefore, we conclude that the observed tradeoff between reproduction and immunity is due to limited capacity for immune-related protein synthesis as a consequence of prior reproductive investment by mated females.

## Discussion

The insect fat body is a highly pleiomorphic tissue that controls key functions including reproduction, immunity, and metabolism. However, how the fat body controls multiple distinct functions is not well understood. In this study, we used single-nucleus RNA-sequencing to demonstrate that the fat body is composed of a collection of heterogeneous cellular subpopulations, each of which appears to have somewhat distinct functionality. Yet we also determined that the entire fat body tissue becomes engaged in the immune response. We unexpectedly discovered that the simultaneous demands of reproductive investment and immune response cause sufficient stress to the fat body that ribogenesis is reduced and protein translation is impaired, including limited production of antimicrobial peptides required to combat infection. This translational stress can be relieved by transiently blocking translation in mated females prior to immune challenge, which presumably frees the translational machinery, restoring antimicrobial peptide production and rescuing survivorship of infection. We propose that this phenomenon may illustrate a general constraint of using the same tissue for multiple functions and that inherent cellular limitations may underly commonly observed tradeoffs such as that between reproduction and immunity.

In addition to revealing global transcriptional patterns, single-nucleus RNA sequencing provides resolution for determining how subsets of cells respond to stimulus and for disaggregating cells originating from distinct tissue types. The fat body of adult *Drosophila* is a fragile and somewhat amorphous tissue, intertwined with multiple other tissues and cell types, including hemocytes, oenocytes, and muscle. This anatomy makes it challenging to dissect and separate the fat body and means that bulk RNA-sequencing will always reflect a composite of these multiple cell types. The advantage of single-nucleus sequencing, as applied here, is that the transcriptionally active nuclei can be computationally inferred as belonging to different tissues, allowing targeted evaluation of how a single tissue responds to stimulus without signal corruption from physically associated cells belonging to other tissues. The present data allow clean discrimination of the fat body from extraneous cell types. Focusing specifically on the fat body, we observe six major cell type clusters that are characterized by distinct transcriptional profiles that suggest distinct biological function. Remarkably, the relative size of the clusters does not change in response to mating or infection treatments, nor across biological replicates, suggesting that the transcriptional clusters represent fixed cellular subpopulations. It seems plausible that cellular differentiation within the fat body may enable the tissue to sustain distinct physiological functions in parallel. Each of these clusters is defined by the expression of a distinct set of marker genes. Future development of reporter constructs reflecting expression of these markers should be developed to address questions relating to the spatial organization and developmental provenance of the cellular subtypes.

Despite the clear and repeatable resolution of the fat body cells into distinct subpopulations, we observed that the entire tissue is highly responsive to infection. All of the fat body clusters strongly induce expression of genes encoding antimicrobial peptides after the bacterial infection treatment. Pseudotime analysis, which arrays cells in two-dimensional space as a function of their transcriptional similarity, completely dissociates the cells from infected versus uninfected animals. The infected and uninfected pseudotime trajectories of fat body clusters are essentially mirror images displaced in transcriptional space, indicating that the structure of cellular heterogeneity is preserved in both states but that the massive tissue-wide transcriptional response to infection (e.g., [10]) is so dramatic that it overrides the dissimilarities that define the cellular subpopulations within the tissue. Notably, mating does not have this same effect, supporting previous observations that the transcriptional response to mating is quantitatively mild in comparison [27] and that mating status does not substantially alter the transcriptional response to infection [28]. Each of the transcriptional clusters corresponding to non-fat-body cells exists in only one of the two pseudotime trajectories, demonstrating that these other cell types have a comparatively relatively limited transcriptional response to mating and infection.

Our transcriptional analysis suggested that a primary difference between mated and virgin flies might be in their translation capacity, based on downregulation of genes involved in ribosome biogenesis in the mated samples. This downregulation was unexpectedly profound in the samples from flies that were both mated and infected (MI). Additionally, we observed transcriptional signatures of ER stress in the MI females, consistent with a previous report that described ER stress upon infection in *D. melanogaster* [29]. Previous studies that used whole flies to interrogate the transcriptional response to mating (e.g., [28, 30]) or infection (e.g., [10]) have not highlighted ribogenesis genes as a category that change expression substantially in response to infection. A probable explanation may be that the suppressed ribogenesis occurs primarily in the fat body and is masked in samples derived from whole flies.

The transcriptional suppression of ribogenesis genes and signatures of ER stress in MI females in particular prompted us to test whether those flies were actually deficient in the capacity to produce protein and whether that deficiency might provide a mechanistic explanation for the commonly observed tradeoff between reproduction and immunity in *D. melanogaster* (e.g., [6, 12, 13]). Consistent with this hypothesis, we found that female *D. melanogaster* were deficient in protein translation specifically after the combined demands of reproductive investment and response to infectious challenge. In addition to a general reduction in protein translation, these females are measurably deficient in their ability to produce antimicrobial peptides in response to infection, which presumably contributes to their susceptibility to infection and drives the tradeoff. The deficiencies in protein production, antimicrobial synthesis, and survival of infection can all be rescued by transiently providing mated females with reversible inhibitor of translation prior to infection, apparently sufficiently freeing the fat body translation machinery to enable a robust immune defense. An unexplained observation in our data, however, is that the fat bodies of mated females appear to produce similar amounts of protein as those of virgin females despite transcriptional indication of reduced ribogenesis. We speculate that the virgin females have more unoccupied ribosomes than the mated females, which allows them to rapidly increase production of proteins including antimicrobial peptides upon infection. In contrast, infection challenge seems to drive mated females into ER stress and tissue collapse, reducing protein synthesis and increasing susceptibility to infection.

The cycloheximide used to reversibly inhibit translation will act on the entire body [31], and our measures of protein synthesis are taken from whole flies. Therefore, our study cannot rule out the possibility that tissues other than fat body may contribute to differential defense against infection in mated versus virgin *D. melanogaster*. However, the fat body is the primary site of antimicrobial peptide production in insects including *D. melanogaster* [32] and therefore differential translation of these peptides in the fat bodies of virgin versus mated females is likely to underlie the difference in antimicrobial peptide abundance we observed by mass spectrometry. The transcriptional signatures of reduced ribogenesis in the fat body and observation of ER stress here and in [29] additionally support a major role for translation in the fat body. Future efforts into characterizing the activity of master regulators of ribosome biogenesis and factors that regulate protein synthesis in cell-specific manners will help define the contributions of tissue-specific and organism-wide translation capacity to the overall quality of immune defense.

The fact that immunity can be partially rescued with cycloheximide treatment suggests the potential for plasticity in the reproduction-immunity tradeoff. Flies could, in theory, sustain greater immune capacity by reducing their commitment to reproductive investment. Thus, genetic variation for reproductive investment could allow natural selection to shape the tradeoff over time and space as a function of infection risk. Reduced translation, specifically in the fat body [33], extends lifespan in *Drosophila* [34] through evolutionarily conserved mechanisms [35] shared with organisms such as *C. elegans* [36, 37] and mice [38], while mating and reproduction are costly and reduce lifespan in fruit flies and other organisms [39]. Translation in the fat body or analogous tissues could therefore additionally be a mechanism mediating reproduction-longevity tradeoffs in insects and other organisms. It seems likely that environmental factors such as amino acid nutrition may also influence the shape of tradeoffs governed by translation.

## Conclusion

Managing competing physiological demands is a critical challenge for any polyfunctional tissue. We find here that the *Drosophila melanogaster* fat body executes diverse basal functions via heterogeneous cellular subpopulations. However, the whole tissue becomes engaged in an immune response. The gene expression markers that we have identified as defining the cellular subpopulations can serve to develop reporters that will enable future research into the dynamism and spatial structure of the *Drosophila* fat body. The fat body is a remarkable tissue that is highly responsive in regulating multiple aspects of physiology. However, while the fat body is enormously flexible, the shared reliance of multiple functions on a single tissue inherently leads to constraints and tradeoffs. As we have shown in defining the protein translation failure that causes a reproduction-immunity tradeoff, compound stresses can overwhelm the tissue and lead to adverse outcomes. Understanding strategies that polyfunctional tissues use for balancing critical functions at the whole-tissue and sub-tissue levels can elucidate general mechanisms of physiological and evolutionary tradeoffs that underpin life history theory.

## Materials and methods

### Fly husbandry

All experiments were performed using four-day post-eclosion *Drosophila melanogaster* females of the strain Canton S. Flies were raised on *ad libitum* cornmeal-sugar-yeast medium containing 6% Brewer’s yeast, 6% Cornmeal, 4% Sucrose, 0.7% Agar, 0.04% phosphoric acid and 0.004% propionic acid at 25°C on 12H:12H Light: Dark cycle.

### Mating treatment

Females in this experiment are either virgin or mated. Virgins were collected by harvesting females within 6 h of eclosion from the pupal case and housing them without males. To generate mated flies, 10 females were combined with 10 males in replicate vials for 24 h prior to downstream experiments. The vials were observed for 30 min after males and females were combined to ensure that mating took place. More than 90% of females began mating within the first 15 min. Males and females were held together for 24 h to ensure that all the females mated.

### Infection treatment

For the infection treatment, both virgin and mated females were infected with the Gram-negative bacterium *Providencia rettgeri*. Mated females were separated from males at the time of infection. To generate the bacterial inoculum, *P. rettgeri* were grown to saturation overnight in Lysogeny Broth (LB) broth at 37°C with shaking (200 rpm). Bacteria from the overnight culture were pelleted and resuspended in phosphate-buffered saline (PBS) to an optical density of A600 = 1.0. Flies were infected while under CO_2_ anesthesia by pricking the thorax with a 0.1 mm diameter minutien pin that had been dipped in the bacterial suspension [40]. This procedure delivers approximately 3000 bacteria to each fly. Uninfected females were handled in exactly the same way except they were not pricked with the needle.

### Post-infection survival assay

To assay survival after infection, females were housed in groups of ten per vial, with the number of dead flies recorded every 24 h for 4 days post-infection. Individuals surviving at the end of four days were censored. Post-infection survival analysis was done using Cox-proportion hazards model in JMP Pro v15. Pairwise risk ratios were calculated to find differences between treatments.

### Structure of the snRNA-seq experiment

The snRNA-seq experiment was replicated twice across the four treatments: Virgin-Uninfected (VU), Virgin-Infected (VI), Mated-Uninfected (MU), and Mated-Infected (MI). All of the treatments were handled independently on separate days. The two replicate experiments were performed two months apart. A separate set of egg collections was done for each treatment. Flies were allowed to mature for four days prior to mating and infection treatments. Fat bodies of infected females were dissected at 6 h post-infection.

### Fat body dissections and nuclei preparation

Single-nuclei were isolated from abdominal fat body and associated tissues using the protocol described in Gupta and Lazzaro [14]. Briefly, 4-day old adults were anesthetized using light CO_2_. The posterior tip of the abdomen was pulled out using fine forceps and the cuticle was cut laterally using spring scissors. The cuticle was carefully opened in cold adult hemolymph-like saline (HLS) and the gut and ovaries were removed. The cuticle and adherent issues were immediately transferred to chilled methanol fixative in order to preserve the quality of the RNA. After pooling tissues from 40 flies, cells were lysed in a hypotonic buffer using a Dounce homogenizer. Suspended nuclei were then purified using series of low-speed and sucrose gradient centrifugation steps. After purification, nuclei were suspended in PBS containing 2% BSA. If cell debris was observed, the nuclei suspension was filtered using a 20-μm cell filter. Nuclei suspended in PBS (+2% BSA) on ice were immediately taken for 10X Chromium platform-based RNA sequencing. In pilot experiments, we found that this protocol dramatically improved sequencing quality and minimized mitochondrial contamination [14].

### 10X Chromium nuclei preparation

A small aliquot of the nuclei sample was incubated with Trypan Blue and the stained nuclei were counted using an automated cell counter. We used the total nuclei count to estimate sample volume required to sequence 7000 nuclei per sample on the 10X platform. RNA libraries were prepared using 10X chromium v3 chemistry and downstream sample processing was done as per the manufacturer’s protocol. Libraries were sequenced on Illumina platform following 10X recommended conditions.

### Data processing and analyses

We used Cell Ranger [40] mkfastq v3.0 (10x Genomics;) to generate demultiplexed FASTQ files from the raw sequencing reads. Reads were aligned to the *Drosophila* genome and quantified gene counts as UMIs using Cell Ranger count v3.0 (10x Genomics). For snRNA-Seq reads, we counted reads mapping to introns as well as exons, as this results in a greater number of genes detected per nucleus, more nuclei passing quality control and better cell type identification, as previously described [41, 42]. To count introns during read mapping, we followed the approach described at https://support.10xgenomics.com/single-cell-gene-expression/software/pipelines/latest/advanced/references. Briefly, we built a “pre-mRNA” *Drosophila* dmel-all-r6.24 reference using Cell Ranger [40] mkref v3.0 (10x Genomics) with a modified gene transfer format (GTF) file Dmel v6 from Flybase. which was then used to align raw Illumina sequence reads to obtain sparse single-cell expression matrix. The resulting matrices were analyzed using R [43] v3.5.3, and Seurat [44, 45] v3.1. After performing sample QC using Seurat [44, 45] and batch correction using Harmony [46] (Fig. S1), we obtained data from 56,000 nuclei across all samples that were further processed to cluster nuclei into subpopulations (resolution 0.5) and identify cluster-specific canonical markers. We used the package “scDblFinder” in R to identify doublets in our dataset. Approximately 5% doublets were identified in each dataset (Table S1A) and they were distributed across all clusters (Table S1B). The number of these doublets is in the expected range for sequencing 7000 nuclei per sample (as provided by 10X Chromium). The Spearman correlation between genes expressed in the two replicates was calculated using package “LSD” in R. Differential gene expression was evaluated using the ‘FindMarkers’ function in Seurat to detect genes that were differentially expressed between treatments in both the replicates (FDR<1%) while not being differentially expressed between the two VU replicates. A Wilcox test was used to calculate *p* values for significance of the difference in expression level.

We used Monocle v3.0 [20, 21, 46] to perform pseudotime analysis on the dataset. Gene modules containing co-varying genes were identified using stringency set to *q* value<0.01. The gene sets obtained from these analyses were used for interpretation by performing Gene Ontology (GO) term enrichment analysis with g:Profiler [47] using default settings (*p*<0.01) for molecular function and biological process. GO: Profiler was also used to determine significantly enriched (*p*<0.01) KEGG pathways.

On the basis of GO term analysis of conserved marker gene expression, we identified six major fat body subpopulations (Clusters 0, 1, 2, 5, 7, and 10) in our dataset. To specifically query the response of fat body cells to different treatments (Fig. S9), we created a dataset containing only the definitively identified fat body cells from those six clusters. We performed pseudotime and GO term analysis on this restricted subset of the data as described earlier.

### Puromycin incorporation assay

We measured global translation in the fat body using puromycin incorporation. Puromycin is an antibiotic which gets incorporated in nascent polypeptides during translation. Incorporated puromycin in polypeptides is then quantified using Western Blotting as per previously published protocols [48, 49]. The experiment was replicated five times. For each replicate of the experiment, ten adult female fat body tissues from each treatment were dissected in ice-cold adult hemolymph-like saline as described above. Tissues were stored on ice in 1 mL Schneider’s medium until ten tissues per sample were dissected and pooled. Tissues were carefully transferred to pre-warmed 1 ml Schneider’s medium containing 10μg/mL puromycin. Samples were incubated at 25°C for 1 h. After the incubation, 330μl of 50% trichloroacetic acid was added. Protein samples were prepared in SDS-PAGE loading buffer after washing tissues with 1M Tris base. Proteins were separated on 12% gel (Bio-Rad Mini-PROTEAN TGX Precast Protein Gels). Puromycin incorporation was assayed using anti-puromycin antibody (Millipore #12D10) and anti-actin antibody (Cell Signaling Technology #D6A8) was used to quantify actin as a loading control. Dual detection was performed using LiCor NIR fluorescent secondary antibodies - IRDye® 680RD Donkey-anti-Rabbit Antibody and IRDye® 800CW Donkey-anti-Mouse Antibody. Quantitative measurements were performed using LiCor Image Studio Lite, and the signal detected for each sample was relativized to its actin intensity. Difference in puromycin signal across treatments was determined using one-way ANOVA in JMP Pro v15 with “treatment” (VU, VI, MU, and MI) as the predictive factor.

### Cycloheximide feeding and survival assays

We fed flies cycloheximide to inhibit translation and assay the effect of translation inhibition on immune defense in mated females. Flies were collected as virgins within 6 h of eclosion and held in single-sex groups of ten flies per vial for 3-4 days. For the cycloheximide treatment, 50μl of 35 mM cycloheximide solution was put on the surface of food in vials and allowed to air dry. A dose of 35 mM has been previously used to inhibit translation in *D. melanogaster* [50–52]. Virgin males and females were randomly chosen and three treatments were set up. In the virgin treatment (VI), females were held in single sex group of ten per vial in four replicate vials. Flies for the mating treatment (MI) were set up by combining 10 males with 10 females per vial in four replicate vials. For cycloheximide treatment (CHX), 10 males and 10 females were directly transferred to each of four replicate vials containing CHX-food. Flies were allowed to feed on CHX for 18 h; after which they were transferred to fresh vials with no CHX to allow flies to recover and clear CHX. Flies recovering within 6–12 h of cycloheximide treatment has been shown in [26]. Six to 7 h later, flies were infected with *P*. *rettgeri* as described above. Three independent replicate blocks were generated following this protocol. The effect of cycloheximide on post-infection translation was assayed using puromycin incorporation and Western blotting as described above. To test for difference in translation between CHX and MI, a paired t-test with treatments paired within replicate blocks was performed in R [41] v3.5.3. Box plots were plotted using package ggpubr [53]  in R v3.5.3.

Our experimental results showed that CHX treatment of mated females improved their ability to survive infection. To validate that immunity was enhanced in mated females by CHX-inhibition of translation only when CHX was delivered after mating but before infection, we performed additional control experiments that included three treatments in addition to the VI, MI, and CHX treatments described above. In the first of these, virgin females were transferred to CHX-containing (CHX-V) food for 18 h, after which they were transferred to fresh vials with no CHX for 6 h prior to infection. Four such vials were set up with ten flies per vial. This treatment mirrors the MI treatment, except the flies are not mated, and it tests whether CHX has a directly protective effect against infection. For the second treatment, flies were fed with CHX **c**ontinuously (CHX-C). In this treatment, females were transferred to CHX-treated food at the time of mating and were maintained on CHX food for the duration of the experiment, including after infection. For the third treatment, females were combined with males on normal food and these flies were transferred on CHX food only post infection with bacteria (CHX-PI). Thus, in this treatment, the females will be fully invested in reproduction and will have translation impaired as they initiate their immune response. These additional treatments were replicated three times with post-infection mortality in all experiments recorded every 24 h for 4 days. The individuals surviving at the end of four days were censored in the analysis. The survivorship data are shown in Fig S10. We found that inhibiting translation prior to mating but returning the flies to food without CHX prior to infection (the original CHX treatment) significantly improves survivorship of infection (*p* < 0.0001) relative to females who are not provided with CHX in the food (MI). However, if CHX is provided continuously (CHX-C) or after infection (CHX-PI), survivorship is the same or worse as is observed for MI flies and is significantly worse in than is observed for VI, CHX-VI, or CHX flies (*p* < 0.0001). There was no difference in survivorship between VI and CHX-VI flies (*p* =0.69). Thus, CHX treatment only promotes survivorship of infection when the CHX limits reproductive investment without impairing immune-related translation.

### Electron microscopy

Following the protocol described for mating and infection as described above, flies for four different treatments (VU, VI, MU, MI) were set-up and 5-10 tissues per treatment were dissected. Tissues were immediately fixed in 2% glutaraldehyde, 2.5% formaldehyde, 0.1 M Na cacodylate, pH 7.4., and then transferred into 2.5% glutaraldehyde in 0.05M cacodylate buffer pH 7.4 for 2 h at 4°C. Samples were post-fixed in 1% OsO_4_, 0.05M cacodylate buffer for 1 h at 4°C. The samples were dehydrated in 25% ethanol (4°C for 15’) and 50% ETOH (4°C for 15’). We placed the samples in 2% uranyl acetate in 70% ETOH at 4°C for 48 h. After 48 h, serial dehydration was conducted in ethanol (first 95% then 100%) followed by two washes with 100% acetone. Both the dehydration and washing steps lasted 10 min each and were done at 4 degrees Celsius. The abdomens were infiltrated with Embed 812 (EMS #14120). The abdomens were then embedded in flat molds and polymerized at 60 degrees Celsius for 24 h. The samples were ultramicrotomed on a Leica Ultracut UTC 7 using a Diatome 6° knife. Sixty- to 70-nm thick sections were mounted on 50 mesh copper grids coated with polyvinyl Butvar/carbon grids. Grids were stained for 15 min with 2% uranyl acetate and rinsed with water. The imaging was done with a 120 Thermo-Fisher Tecnai T12 BioTwin at 1200 kV. Images were obtained using a Gatan 794 CCD camera.

### MALDI-TOF data collection and analysis

Flies were collected and treated as described in the above sections describing fly husbandry, mating, infection and cycloheximide treatment. Hemolymph was collected from Virgin-Infected (VI), Mated-Infected (MI), and cycloheximide-treated Mated-Infected females (CHX). Hemolymph was collected 16 h post-infection to allow time for transcription, translation, and secretion of immune molecules into the hemolymph. Decapitated flies were gently pressed at the abdomen and the protruding drop of hemolymph was collected by capillary action into a 1-μl microcap micropipette. Hemolymph from three flies per treatment was collected and immediately transferred to 10 μl of 0.1% trifluoro-acetic acid (TFA) in water. Seven such samples per treatment were collected and samples were stored at − 20°C until MALDI-TOF mass spectrometry. For each MALDI-TOF run, 1 μl of hemolymph sample was mixed with 1 μl of α-cyano-4-hydroxycinnamic acid matrix (CHCA, 25mg/ml in 3:1 Acetonitrile: TFA solution) directly on the MALDI target plate. Samples were analyzed as described previously in [54]. MALDI-TOF mass spectra were acquired using a Bruker AutoFlex MAX MALDI-TOF (Bruker Biospin, Billerica, MA) mass spectrometer. The spectra were acquired in positive-ion reflectron mode (m/z range 1000–6000). Mass ranges were calibrated using a mixture of PEGs 1000, 2000, and 3000 for reflectron mode. Acceleration voltage was set to 20 kV. Five thousand individual laser shots were summed to give the final spectrum. Raw spectra were baseline subtracted and Savitzky-Golay smoothing [55] was applied using mMass [56]. Peaks with a signal/noise ratio > 5 were considered as positive detections. Representative spectra from three treatments are shown in Additional file 1: Fig.S11. To determine the effect of mating and cycloheximide treatments on immune peptides, we counted the number of distinct peptides that were detectable in each of the 7 samples for each of the 3 treatments (21 samples total). Detected peaks could be assigned to known immune-related peptides described previously [27, 36, 54, 57]. Differences between treatments were evaluated using paired *t*-tests as implemented in R v3.5.

## Supplementary Information

**Additional file 1: Fig. S1.** Quality Control of single-nuclei sequencing data of fat body cells of *Drosophila melanogaster*. **Fig. S2.** Metrics of single-nuclei sequencing data from D. *melanogaster* fat body. **Fig. S3.** UMAP of expression of cluster-specific markers. **Fig. S4.** Spatial localization of Cluster 3. **Fig. S5.** Frequency distribution of genes that are differentially expressed between treatments across six fat body clusters. **Fig. S6.** Heatmaps of top differentially expressed genes. **Fig. S7.** Trajectory analysis profiling of *Drosophila* fat body tissue. **Fig. S8.** Enrichment plot of genes expressed in Module 13 obtained in pseudotime analysis. **Fig. S9.** Trajectory analysis profiling of subset of clusters identified as the fat body subpopulations (Clusters 0, 1,2,5,7 and 10). **Fig. S10.** Analysis of Module 15 (Trajectory 2) showing ER stress. **Fig. S11.** Survivorship analysis of post-infection mortality. **Table S1.** Summary of number of doublets identified. **Table S2.** Summary of markers identified for each cluster. **Table S3.** Summary of differentially expressed genes per cluster. **Table S4.** ANOVA summary of puromycin incorporation. **Table S5.** *t-test* results of effect of cycloheximide treatment on puromycin incorporation. **Table S6**. *t-test* results comparing number of peptides detected in MALDI-TOF.

**Additional file 2. **Differentially expressed genes upon mating. padj = *P*-value adjusted for multiple testing. fc = log fold-change between the two treatments. Pct. 1 = percentage of cells in Mated-Uninfected (MU) expressing the gene. Pct. 2 = percentage of cells in Virgin-Uninfected (VU) expressing the gene.

**Additional file 3. **Enrichment analysis of six identified fat body clusters 0,1,2,5,7, and 10 using genes differentially expressed between Mated-Uninfected (MU) and Virgin-Uninfected (VU). adjusted_*p*_value = *P* value adjusted for multiple testing.

**Additional file 4.** Differentially expressed genes upon infection in virgins. padj = P-value adjusted for multiple testing. fc = log fold-change between the two treatments. Pct. 1 = percentage of cells in Virgin-Infected (VI) expressing the gene. Pct. 2 = percentage of cells in Virgin-Uninfected (VU) expressing the gene.

**Additional file 5. **Differentially expressed genes upon infection in mated females. padj = *P*-value adjusted for multiple testing. fc = log fold-change between the two treatments. Pct. 1 = percentage of cells in Mated Infected (MI) expressing the gene. Pct. 2 = percentage of cells in Mated Uninfected (MU) expressing the gene. adjusted_*p*_value = *P*-value adjusted for multiple testing.

**Additional file 6. **VI-VU: Enrichment analysis of six identified fat body clusters 0,1,2,5,7, and 10 using genes differentially expressed between Virgin-Uninfected (VU) and Virgin-Infected (VI)**.** MI-MU: Enrichment analysis of six identified fat body clusters 0,1,2,5,7, and 10 using genes differentially expressed between Mated Uninfected (MU) and Mated Infected (MI). adjusted_*p*_value = *P*-value adjusted for multiple testing.

**Additional file 7.** Genes expressed in module 13 of partition 1. Genes expressed in this module show low aggregate gene expression score in Mated Uninfected.

**Additional file 8.** Genes expressed in module 16 of partition 2. Genes expressed in this module show low aggregate gene expression score in Mated Infected.

**Additional file 9.** Genes expressed in module 15 of partition 2. Genes expressed in this module show high aggregate gene expression score in Mated Infected.

**Additional file 10. **Marker genes for each cluster (Clusters 0 to 18) obtained using function ‘FindConservedMarkers’ in Seurat. Avg_logFC: average log fold change between cluster of interest and all other clusters. minimum p_val: combined *p*-value for all the four treatments (Virgin-Uninfected. Virgin Infected, Mated Uninfected, Mated Infected).

**Additional file 11.** Enrichment analysis of marker genes for each cluster (Clusters 0 to 18).

## Data Availability

All sequence data generated for this study are available in the NCBI Short Read Archive, accession number PRJNA698971 [58].

## References

[1] Roff DA. Life history evolution. United Kingdom: Sinauer; 2002.

[2] Stearns SC. The Evolution of Life Histories. United Kingdom: Oxford University Press; 1992.

[3] Sheldon BC, Verhulst S (1996). Ecological immunology: costly parasite defences and trade-offs in evolutionary ecology. Trends Ecol Evol.

[4] Schwenke RA, Lazzaro BP, Wolfner MF (2016). Reproduction–Immunity Trade-Offs in Insects. Annu Rev Entomol.

[5] Norris K, Evans MR (2000). Ecological immunology: Life history trade-offs and immune defense in birds. Behav Ecol.

[6] Schwenke RA, Lazzaro BP (2017). Juvenile Hormone Suppresses Resistance to Infection in Mated Female *Drosophila melanogaster*. Curr Biol.

[7] Arrese EL, Soulages JL (2010). Insect fat body: energy, metabolism, and regulation. Annu Rev Entomol.

[8] Li S, Yu X, Feng Q (2019). Fat body biology in the last decade. Annu Rev Entomol.

[9] De Gregorio E, Spellman PT, Rubin GM, Lemaitre B (2001). Genome-wide analysis of the *Drosophila* immune response by using oligonucleotide microarrays. Proc Natl Acad Sci U S A.

[10] Clark RI, Tan SWS, Péan CB, Roostalu U, Vivancos V, Bronda K (2013). XMEF2 is an in vivo immune-metabolic switch. Cell..

[11] Segerstrom SC (2007). Stress, energy, and immunity: An ecological view. Curr Dir Psychol Sci.

[12] Short SM, Wolfner MF, Lazzaro BP (2012). Female *Drosophila melanogaster* suffer reduced defense against infection due to seminal fluid components. J Insect Physiol..

[13] Fedorka KM, Linder JE, Winterhalter W, Promislow D (2007). Post-mating disparity between potential and realized immune response in *Drosophila melanogaster*. Proc R Soc B Biol Sci..

[14] Gupta V, Lazzaro BP (2022). A robust method to isolate *Drosophila* fat body nuclei for transcriptomic analysis. Fly (Austin).

[15] Droujinine IA, Perrimon N (2016). Interorgan Communication Pathways in Physiology: Focus on *Drosophila*. Annu Rev Genet.

[16] Rajan A, Perrimon N (2011). *Drosophila* as a Model for Interorgan Communication: Lessons from Studies on Energy Homeostasis. Dev Cell.

[17] Bloch Qazi MC, Heifetz Y, Wolfner MF (2003). The developments between gametogenesis and fertilization: Ovulation and female sperm storage in *Drosophila melanogaster*. Dev Biol.

[18] Dionne MS (2014). Immune-metabolic interaction in *Drosophila*. Fly (Austin).

[19] Trapnell C, Cacchiarelli D, Grimsby J, Pokharel P, Li S, Morse M (2014). The dynamics and regulators of cell fate decisions are revealed by pseudotemporal ordering of single cells. Nat Biotechnol.

[20] Qiu X, Hill A, Packer J, Lin D, Ma YA, Trapnell C (2017). Single-cell mRNA quantification and differential analysis with Census. Nat Methods.

[21] Qiu X, Mao Q, Tang Y, Wang L, Chawla R, Pliner HA (2017). Reversed graph embedding resolves complex single-cell trajectories. Nat Methods.

[22] Back SH, Kaufman RJ (2012). Endoplasmic reticulum stress and type 2 diabetes. Annu Rev Biochem.

[23] Walter P, Ron D (2011). The Unfolded Protein Response: From Stress Pathway to Homeostatic Regulation. Science (80- ).

[24] Ellner SP, Buchon N, Dörr T,Lazzaro BP. Host–pathogen immune feedbacks can explain widely divergent outcomes from similar infections. Proc R Soc B. 2021;288:20210786. 10.1098/rspb.2021.0786 PMC8150042 34034518

[25] David D, Jean-Baptiste F, Jonathan R, Hannah K, Ortiz Gerardo A, Lazzaro BP, Buchon N (2017). Stochastic variation in the initial phase of bacterial infection predicts the probability of survival in D. *melanogaster*. Elife.

[26] Bownes M, Scott A, Blair M (1987). The use of an inhibitor of protein synthesis to investigate the roles of ecdysteroids and sex-determination genes on the expression of the genes encoding the *Drosophila* yolk proteins. Development..

[27] Carboni AL, Hanson MA, Lindsay SA, Wasserman SA, Lemaitre B. Cecropins contribute to *Drosophila* host defense against a subset of fungal and Gram-negative bacterial infection. Genetics. 2022;220(1):iyab188. 10.1093/genetics/iyab188 PMC8733632 34791204

[28] Short SM, Lazzaro BP (2013). Reproductive status alters transcriptomic response to infection in female *Drosophila melanogaster*. G3 Genes, Genomes, Genet.

[29] Troha K, Im JH, Revah J, Lazzaro BP, Buchon N. Comparative transcriptomics reveals CrebA as a novel regulator of infection tolerance in D. *melanogaster*. PLoS Pathog. 2018;14:e1006847. 10.1371/journal.ppat.1006847 PMC5812652 29394281

[30] Rodrigues MA, Merckelbach A, Durmaz E, Kerdaffrec E, Flatt T (2021). Transcriptomic evidence for a trade-off between germline proliferation and immunity in *Drosophila*. Evol Lett.

[31] Schneider-Poetsch T, Ju J, Eyler DE, Dang Y, Bhat S, Merrick WC (2010). Inhibition of eukaryotic translation elongation by cycloheximide and lactimidomycin. Nat Chem Biol.

[32] Lemaitre B, Hoffmann J (2007). The Host Defense of *Drosophila melanogaster*. Annu Rev Immunol.

[33] Tain LS, Sehlke R, Jain C, Chokkalingam M, Nagaraj N, Essers P (2017). A proteomic atlas of insulin signalling reveals tissue-specific mechanisms of longevity assurance. Mol Syst Biol.

[34] Wang D, Cui Y, Jiang Z, Xie W (2014). Knockdown expression of eukaryotic initiation factor 5 C-terminal domain containing protein extends lifespan in *Drosophila melanogaster*. Biochem Biophys Res Commun.

[35] McElwee JJ, Schuster E, Blanc E, et al. Evolutionary conservation of regulated longevity assurance mechanisms. Genome Biol. 2007;8R132. 10.1186/gb-2007-8-7-r132. 10.1186/gb-2007-8-7-r132 PMC2323215 17612391

[36] Hansen M, Taubert S, Crawford D, Libina N, Lee SJ, Kenyon C (2007). Lifespan extension by conditions that inhibit translation in Caenorhabditis elegans. Aging Cell.

[37] Pan KZ, Palter JE, Rogers AN, Olsen A, Chen D, Lithgow GJ (2007). Inhibition of mRNA translation extends lifespan in *Caenorhabditis elegans*. Aging Cell.

[38] Thompson ACS, Bruss MD, Price JC, Khambatta CF, Holmes WE, Colangelo M (2016). Reduced in vivo hepatic proteome replacement rates but not cell proliferation rates predict maximum lifespan extension in mice. Aging Cell.

[39] Thomas F, Andreas H. Mechanisms of Life History Evolution: The Genetics and Physiology of Life History Traits and Trade-Offs. United Kingdom: OUP Oxford.; 2011. 10.1093/acprof:oso/9780199568765.001.0001.

[40] Khalil S, Jacobson E, Chambers MC, Lazzaro BP. Systemic Bacterial Infection and Immune Defense Phenotypes in *Drosophila Melanogaster*. J Vis Exp. 2015;(99):e52613. 10.3791/52613. 10.3791/52613 PMC4542538 25992475

[41] Bakken TE, Hodge RD, Miller JA, Yao Z, Nguyen TN, Aevermann B (2018). Single-nucleus and single-cell transcriptomes compared in matched cortical cell types. PLoS One.

[42] Zheng G, Terry J, Belgrader P, et al. Massively parallel digital transcriptional profiling of single cells. Nat Commun. 2017;8:14049. 10.1038/ncomms14049 PMC5241818 28091601

[43] R Core Team. R (2019). A language and environment for statistical computing. R Foundation for Statistical Computing, Vienna, Austria.

[44] Butler A, Hoffman P, Smibert P, Papalexi E, Satija R (2018). Integrating single-cell transcriptomic data across different conditions, technologies, and species. Nat Biotechnol.

[45] Stuart T, Butler A, Hoffman P, Hafemeister C, Papalexi E, Mauck WM (2019). Comprehensive Integration of Single-Cell Data. Cell..

[46] Korsunsky I, Millard N, Fan J, Slowikowski K, Zhang F, Wei K (2019). Fast, sensitive and accurate integration of single-cell data with Harmony. Nat Methods.

[47] Raudvere U, Kolberg L, Kuzmin I, Arak T, Adler P, Peterson H (2019). G:Profiler: A web server for functional enrichment analysis and conversions of gene lists (2019 update). Nucleic Acids Res.

[48] Martínez Corrales G, Filer D, Wenz KC, Rogan A, Phillips G, Li M (2020). Partial Inhibition of RNA Polymerase I Promotes Animal Health and Longevity. Cell Rep.

[49] Deliu LP, Ghosh A, Grewal SS (2017). Investigation of protein synthesis in *Drosophila* larvae using puromycin labelling. Biol Open.

[50] Krause T, Spindler L, Poeck B, Strauss R (2019). *Drosophila* Acquires a Long-Lasting Body-Size Memory from Visual Feedback Report *Drosophila* Acquires a Long-Lasting Body-Size Memory from Visual Feedback. Curr Biol.

[51] Widmann A, Artinger M, Biesinger L, Boepple K, Peters C, Schlechter J (2016). Genetic Dissection of Aversive Associative Olfactory Learning and Memory in *Drosophila* Larvae.

[52] Ge X, Hannan F, Xie Z, Feng C, Tully T, Zhou H (2004). Notch signaling in *Drosophila* long-term memory formation.

[53] Kassambara A (2019). ggpubr: “ggplot2” Based Publication Ready Plots. R package version 0.2.4.

[54] Bulet P, Uttenweiler-Joseph S. A MALDI-TOF Mass Spectrometry Approach to Investigate the Defense Reactions in *Drosophila melanogaster*, an Insect Model for the Study of Innate Immunity. Proteome Protein Anal. 2000:157–74.

[55] Savitzky A, Golay MJE (1964). Smoothing and Differentiation. Anal Chem.

[56] Niedermeyer THJ, Strohalm M. mMass as a Software Tool for the Annotation of Cyclic Peptide Tandem Mass Spectra. PLoS ONE. 2012;7(9):e44913. 10.1371/journal.pone.0044913. 10.1371/journal.pone.0044913 PMC3441486 23028676

[57] Levy F, Rabel D, Charlet M, Bulet P, Hoffmann JA, Ehret-Sabatier L (2004). Peptidomic and proteomic analyses of the systemic immune response of *Drosophila*. Biochimie..

[58] Single-nucleus sequencing of Drosophila adult fat body tissue (2021). NCBI BioProject accession: PRJNA698971.

