# Supplementary material for: Inherent constraints on a polyfunctional tissue lead to a reproduction-immunity tradeoff
Source: BMC Biol. 2022 Jun 2;20:127. doi: 10.1186/s12915-022-01328-w (PMC9161490; doi:10.1186/s12915-022-01328-w)
Supplement: Supplementary file 1 — Additional file 1: Fig. S1. Quality Control of single-nuclei sequencing data of fat body cells of Drosophila melanogaster. Fig. S2. Metrics of single-nuclei sequencing data from D. melanogaster fat body. Fig. S3. UMAP of expression of cluster-specific markers. Fig. S4. Spatial localization of Cluster 3. Fig. S5. Frequency distribution of genes that are differentially expressed between treatments across six fat body clusters. Fig. S6. Heatmaps of top differentially expressed genes. Fig. S7. Trajectory analysis profiling of Drosophila fat body tissue. Fig. S8. Enrichment plot of genes expressed in Module 13 obtained in pseudotime analysis. Fig. S9. Trajectory analysis profiling of subset of clusters identified as the fat body subpopulations (Clusters 0, 1,2,5,7 and 10). Fig. S10. Analysis of Module 15 (Trajectory 2) showing ER stress. Fig. S11. Survivorship analysis of post-infection mortality. Table S1. Summary of number of doublets identified. Table S2. Summary of markers identified for each cluster. Table S3. Summary of differentially expressed genes per cluster. Table S4. ANOVA summary of puromycin incorporation. Table S5. t-test results of effect of cycloheximide treatment on puromycin incorporation. Table S6. t-test results comparing number of peptides detected in MALDI-TOF. [file 12915_2022_1328_MOESM1_ESM.docx]

Supplementary Materials for

Additional File 1 for

**Inherent constraints on a polyfunctional tissue lead to a reproduction-immunity tradeoff**

Vanika Gupta*, Ashley M. Frank, Nick Matolka, Brian P. Lazzaro^*^

*Corresponding Author: Email:

bplazzaro@cornell.edu, vg272@cornell.edu

**This file includes:**

Supplementary Text

Figs. S1 to S11

Tables S1, S2, S3, S4, S5

Supplementary Text

***Cluster Descriptions***

We performed single nucleus RNA-sequencing on 56,000 nuclei from eight samples (two replicates each of VU, VI, MU and MI treatments). After confirming sequence quality, we performed batch correction using Harmony with default parameters (Figs.S1,2), and clustering using Louvain algorithm with resolution set to 0.5 and default parameters using Seurat (v3.1). This yielded 19 distinct nuclear (cell) subpopulations. The subpopulations are numbered from 0 to 18 in descending order of size, the largest being “Cluster 0” and the smallest being “Cluster 18”.

We performed differential gene expression analysis for each cluster against all other clusters using the function FindConservedMarkers in Seurat to identify genes that were significantly upregulated (FDR <1%) or overexpressed in each cluster across all the four treatments (VU, VI, MU, and MI) when compared to nuclei in all other clusters (Fig.S3, Table S1). These significantly upregulated genes are also called “canonical markers” or “marker genes” or “markers” for each cluster. Next, we performed GO (Gene Ontology) term enrichment (p<0.01) analysis on each list of “marker genes” corresponding to each cluster using g:Profiler [1]**.** We inferred the function sub-type of each of the clusters based on enrichment of GO terms associated biological processes, molecular function, and cellular component along with KEGG pathway enrichment. Some clusters, as expected, appear to belong to cells of other tissues that were co-dissected with the fat body. These expressed distinctive marker genes diagnostic of cell types such as muscle, oenocytes, hemocytes, and crystal cells. We annotated the remaining clusters as fat body subtypes. Below we discuss each cluster in terms of its size (in terms of fraction of total nuclei in the tissue), key marker genes expressed (Additional File 10), and significant GO terms (Additional file 11), and we summarize these findings by inferring a putative function for each cluster.

**Cluster 0 – Fat body cells**

Cluster 0 is the primary cluster containing fat body cells and contains 25% of all the nuclei sequenced. This is the largest and most generic cluster of fat body cells and it has the fewest distinctively expressed marker genes, with only 7 diagnostic marker genes (Table S13). The genes *yolk protein* 3 (*yp3*) and *yolk protein* (*yp1)* are the top two markers expressed in the cluster. Yolk proteins in *Drosophila* are expressed and secreted by the fat body for their uptake by mature oocytes [2]. Additionally, we also found that *Phosphoglucose mutase 1* (*Pgm1*), an enzyme involved in glycolysis, was one of the marker genes. Enriched GO terms included phospholipase A1 activity (Table S14). We conclude that the primary roles of Cluster 0 are producing yolk proteins and metabolism.

**Cluster 1 – Fat body cells**

Cluster 1 consists of 12% of all nuclei sequenced and is defined by 77 markers (Table S13). The large number of marker genes indicates that this cluster may be a specialized sub-type of the fat body tissue. *Heat shock protein 27 (Hsp27)* and *deadhead (dhd)* are the top markers expressed in this cluster. Enriched GO terms included RNA binding, organelle organization, regulation of metabolic and cellular processes (Table S14). The modENCODE bulk RNAseq dataset represented in FlyBase ([www.flybase.org](http://www.flybase.org)) indicates that nine out of the top ten marker genes showed moderately high (minimum RPKM = 29) to very high expression (minimum RPKM = 110) in four-day old carcass. Interestingly, the modENCODE bulk RNAseq dataset showed that these genes are also expressed in the in ovaries, although the ovaries were removed in our tissue dissections. Fat body Cluster 1 may have a role in reproduction.

**Cluster 2 – Fat body cells**

Cluster 2 consists of 11% of nuclei sequenced and was marked by 23 genes (Table S13). The genes *yp3*, *yp1*, and *trehalose-6-phosphate synthase 1* (*Tps1)* were the markers with the highest expression. The top markers of cluster 0 (*yp1*, *yp3* and *Pgm1*) also marked cluster 2. The GO enrichment term “lipase activity” characterized Cluster 2 (Table S14) and KEGG pathways for starch, sucrose, and galactose metabolism were significantly enriched in expression (Table S14). This suggests that, like Cluster 0, cluster 2 is a fat body cluster that has metabolic functions and is involved in reproductive provisioning.

**Cluster 3 – Unknown cell type**

Cluster 3 consists of 10% of sequenced nuclei and distinctively expressed 284 marker genes (Table S13). Top markers of cluster 3 are *Epidermal growth factor receptor* (*Egfr*), *nicotinic acetylcholine Receptor α7* (*nARCH7α*), and *Death-associated protein kinase related (Drak*). Cluster 3 was also marked by expression of genes in the Toll (*Dif*, *Tl*), *Imd* and JAK/STAT (*Stat92E*), and insulin signaling (*thor*, *foxo*) which are immune-related and metabolic pathways. Cluster 3 also expressed as a marker gene *adiponectin receptor* (*AdipoR*), which controls insulin signaling to regulate metabolism and maintain germ-line stem cell populations. Enriched GO terms included biological processes such as response to stimulus and positive regulation of biological process (Table S14). KEGG pathway analysis showed enrichment for purine metabolism (Table S14). Availability of *nARCH7α-*GAL4 driver [3] allowed us to spatially resolve cluster 3 (Fig.S4). Surprisingly, expression of the fluorescent reporter mCherry under the control of nARCH*7a* driver coincided with the location and morphology of pericardial cells [4, 5]. Therefore, although this cluster appears to be involved in immunity and metabolism and has expression patterns indicative of a fat body subpopulation, the physical location and morphology suggest pericardial cells. In the absence of definitive certainty, we conservatively consider this cell type to have an unknown tissue identity.

**Cluster 4 – Muscle cells**

Cluster 4 consists of 7% of all nuclei sequenced and is defined by 277 marker genes (Table S13). The top two most expressed genes of the cluster are *bent (bt),* and *sallimus (Sls)*. Enriched GO terms included biological processes related to striated muscle cell development, muscle cell differentiation, and actomyosin structure organization (Table S14). Therefore, we marked cluster 4 as muscle cells.

**Cluster 5 – Proliferative fat body cells**

Cluster 5 consists of 7% of the sequenced nuclei and was marked by expression of 184 genes (Table S13). Genes *megalin (mgl)* and *CG14661* were the top markers of this cluster. *Megalin* is a glycoprotein and regulates endocytosis [6]. Marker gene *grainyhead* expressed in cluster 5 is a transcription factor of FGFR signaling pathway which promotes wound healing [7], cell proliferation, and cell growth; *grainyhead* has been previously implicated in determining tolerance of bacterial infection [8]. Transcription factor *vvl* was a marker for this Cluster 5 that has been previously shown to upregulate expression of the antimicrobial peptide gene *cecropin* expression in the fat body [9]. Enriched GO terms included (Table S14) biological processes associated with multicellular organism development. The hippo signaling pathway, as well as phenylalanine and tyrosine metabolism were significantly enriched KEGG pathways (Table S14). Expression of *grainyhead* and genes in the hippo pathway suggests that the cells in Cluster 5 may be proliferative [10].

**Cluster 6 – Oenocytes**

Cluster 6 consisted of 7% of nuclei and expressed 124 marker genes (Table S13). The top genes marking the cluster are *FASN2* and *CG7910*. When comparing the markers of this cluster with previously published gene lists enriched in oenocytes and fat body, we found that 37 out of 124 marker genes overlapped with previously published oenocyte-expressed genes [11]. Enriched GO terms included biological processes related to fatty acid metabolic process (Table S14). Since oenocytes in *Drosophila* are primarily responsible for fatty acid biosynthesis [11] and there is high overlap between this cluster and previously defined oenocyte markers, we conclude cluster 6 nuclei are from oenocytes.

**Cluster 7 – Reproductive provisioning fat body cells**

Cluster 7 contained 6% of the nuclei sequenced. Cluster 7 was marked by expression of 617 genes (Table S13). *Oskar (Osk)*, *deadhead (dhd*), and *yolkless* (*yl*) are the top marker genes of cluster 7. Enriched GO terms included enrichment of the biological processes’ terms cellular process, cellular component organization, sexual reproduction and many others (Table S14). Enriched KEGG pathways included phagosome and mTOR signaling pathway. These expression patterns indicate that that cluster 7 is a fat body subpopulation whose functions include reproduction.

**Cluster 8 – Hemocytes**

Cluster 8 was formed by 5% of the sequenced nuclei and was marked by expression of 208 marker genes (Table S13) including *hemolectin (*hml), *tenascin major (Ten-m)*, and *papillin (ppn)*. Enriched GO terms included biological processes related to anatomical structure and animal organ morphogenesis, and enriched KEGG pathways included ECM-receptor interaction, MAPK signaling pathway, and lysosome activity (Table S14). *Hemolectin* is a diagnostic marker [12] of hemocytes and lysosomal activity is a well-characterized phagocyte function so we labelled cluster 8 as hemocytes. Hemocytes have previously been shown to be in close physical association with the fat body, where they act as sentinels of infection and promote expression of immune response genes by the fat body [13].

**Cluster 9 – Uncharacterized**

Cluster 9 consisted of 2% of sequenced nuclei and expressed 877 marker genes (Table S13). *Hsp27* and *wispy (wsp*) are the top two markers of cluster 9. GO enrichment analysis showed that about 400 genes out of 877 were enriched for biological process related to biogenesis and regulation of cellular processes (Table S14). 227 genes out of 877 genes were enriched for reproduction (Table S14). KEGG pathways enriched are Ubiquitin-mediated proteolysis, NOTCH signaling, RNA transport and spliceosome (Table S14). However, we could not interpret any definitive functional pattern. Cluster 9 therefore remains uncharacterized in our dataset.

**Cluster 10 – Structural fat body cells**

Cluster 10 consists of 2% of sequenced nuclei and expresses 54 marker genes (Table S13). Top markers of the cluster included vitelline membrane proteins (*Vm24Aa*, *Vm34Ac*), and *trol*. Laminins (*Lan-A*), perlecan (*trol*) along with heparan sulphate proteoglycan (HSPG) were also markers for this cluster. Together, these genes form CIVICs (Collagen IV Intercellular Concentrations) in the basement membrane of the fat body [14]. CIVICs are a key to inter-adipocyte adhesion in the fat body [14]. Enriched GO terms included anatomical structure development, cell development and differentiation (Table S14). Extra-Cellular Matrix and ECM-receptor interaction was significantly enriched as a KEGG pathway. We concluded that cluster 10 plays a role in maintaining structure of the fat body.

**Cluster 11 – Chorion producing fat body cells**

Cluster 11 consists of 1.9% nuclei of total nuclei. Ten genes marked the cluster 11, seven of which express chorion proteins (Table S13). The top markers of cluster 11 are *Chorion protein 36 (Cp36*) and *Chorion protein 38 (Cp38*). Enriched GO terms included biological process: chorion-containing eggshell formation (Table S14). Dobson et al [15] (2016) reported expression of chorion proteins in tissues other than ovaries. This cluster probably represents a subpopulation of fat body tissue producing chorion proteins for transport to developing oocytes.

**Cluster 12 – Catabolic fat body cells**

Cluster 12 consists of 1.5% of the sequenced nuclei and was marked by 55 expressed genes (Table S13). *Mucin-related-18B* (*Mur18B*) and *CG14292* are the top markers of cluster 12. Enriched GO terms included biological processes associated with ion transport (Table S14). Enriched KEGG pathways were phagosome, oxidative phosphorylation, and metabolic pathways (Table S14). Several genes expressed in these cells encoded vacuolar ATPases (V-ATPases) which have functions in phagocytosis, lysosomal activity, and participate in the mTOR pathway. In larval fat body cells, V-ATPases has previously been shown to play a role in lysosomal formation, acidification and cargo degradation in larval fat body [16]. These cells could be playing a similar role in in the adult fat body as well.

**Cluster 13 – Putative digestive fat body cells**

Cluster 13 consisted of 1% of sequence nuclei and was marked by expression of nine genes (Table S13). *α-Trypsin (αTry)* and *Jonah 65Aiv (jon65Aiv)* are the top marker genes expressed in this cluster. Enriched GO terms included proteolysis biological process. This cluster also expressed three Jonah proteins (Table S14). Although Jonah proteins are highly expressed in the adult midgut [17], their expression has also been reported in larval fat body [18, 19] and fly head [20]. A similar cluster marked by *αTry* and Jonah proteins was previously reported in single nucleus sequencing of male fat bodies and was termed as a putative digestive cell cluster [21]. We retain that notation for cluster 13.

**Cluster 14 – Crystal cells**

Cluster 14 consists of 0.7% of the sequenced nuclei and was marked by expression of 79 genes (Table S13). The top two markers of cluster 14 are *prophenoloxidase 1 (PPO1*) and *prophenoloxidase 2* (*PPO2*)*.* Enriched GO terms included embryo, reproductive structure, and reproductive system development (Table S14). ECM-receptor interaction was the enriched KEGG pathway (Table S14). Given that *PPO1 and PPO2* are predominantly expressed in crystal cells [22], a comparatively rare hemocyte type, we infer cluster 14 to be crystal cells.

**Cluster 15 – Neuronal**

Cluster 15 consists of 0.6% of sequenced nuclei and is marked by expression of 168 marker genes (Table S13). Highly expressed markers of the clusters include *paralytic (para)*, *shaker*, *resistant to dieldrin* (*rdl*). GO term analysis showed enrichment of biological processes such as anterograde trans-synaptic signaling, synaptic signaling, and nervous system development (Table S14). The predominant function associated with Cluster 15 was neurotransmission and we conclude that this cluster represents neuronal cells.

**Cluster 16 – Stress-response fat body cells**

Cluster 16 consists of 0.6% of sequenced nuclei and was marked by four transmembrane proteins: *what else (whe*), *la costa (lcs*), *CG45080, and CG16826* (Table S13). We did not find enrichment for GO terms using the four marker genes of this cluster (Table S14). *Lcs* expression is reported in larval fat body [23]. *Whe* and *lcs* mediate Cyclin J-dependent gut recovery upon bacterial digestion in fruit flies [24]. Therefore, this cluster may play an important role in alleviating stress response possibly including bacterial infection.

**Cluster 17 – Nephrocytes**

Cluster 17 consists of 0.5% of sequenced nuclei (Table S13). The cluster showed expression of 38 marker genes. *Cubilin* and *CG42255* are the top marker genes. GO term analysis (Table S14) showed that the genes expressed in this cluster were enriched for biological processes in nephrocyte diaphragm assembly and nephrocyte filtration, suggesting that this cluster represents nephrocytes.

**Cluster 18 – Tracheal cells**

Cluster 18 consists of 0.4% of sequenced nuclei with 122 genes marking cluster 18 (Table S13). The top two markers expressed in cluster 18 are *antennapedia* (*Antp*) and *waterproof (wat)*. GO term analysis showed expression of *Waterproof* along with 17 other genes (Table S14) enriched for biological process open tracheal development. This suggests Cluster 18 to be tracheal cells.

A


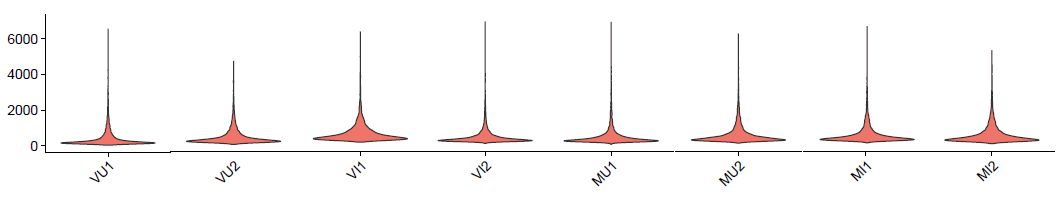


B

D

C

Combined

Genes

E


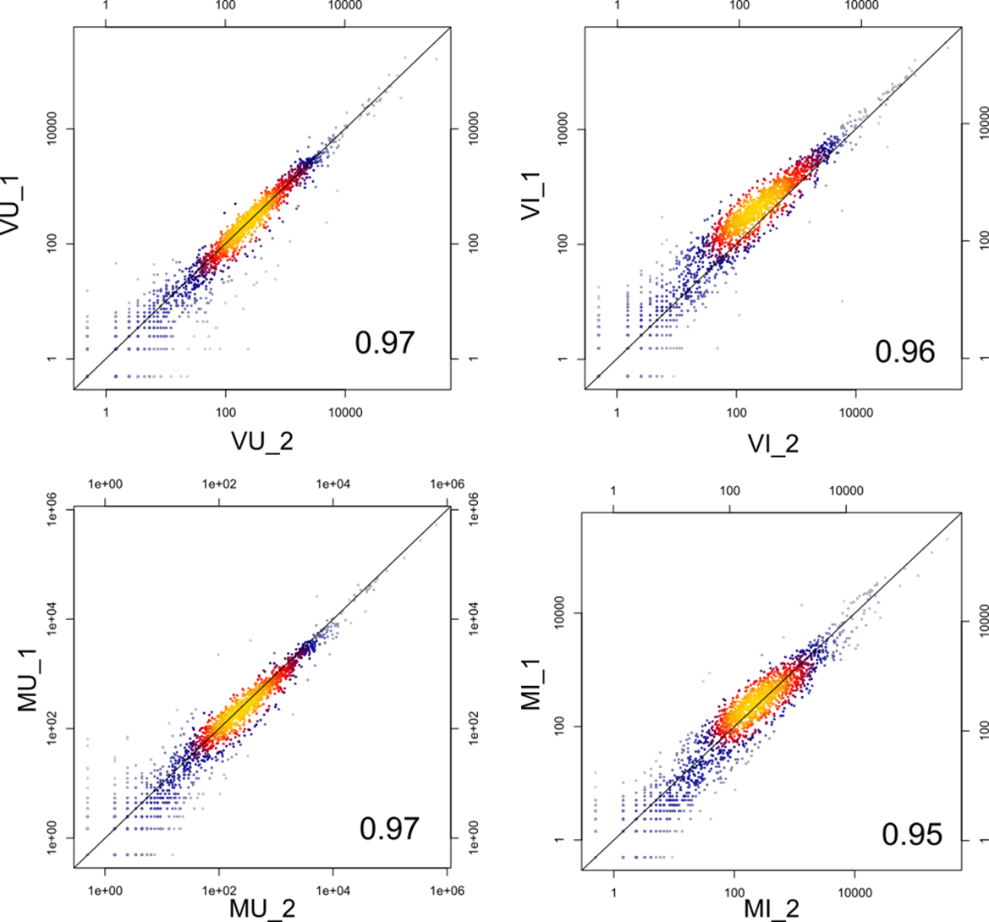

**Before Batch Correction**

F

**After Batch Correction**

Dim 1

Dim 2

G

Dim 1

Dim 2

**Fig.S1.** **Quality Control of single-nuclei sequencing data of fat body cells of *Drosophila melanogaster*.**

In the plots, VU (**V**irgin **U**ninfected), VI (**V**irgin **I**nfected), MU (**M**ated **U**ninfected), MI (**M**ated **I**nfected) are the four treatment groups. Treatment replicates are indicated by 1 and 2. (A) Distribution of the number of genes detected per sequenced nucleus for each sample. (B) Distribution of the number of genes detected per sequenced nucleus across the entire experiment. (C) PCA plot of gene expression data showing first two principal components showing that there are no clear outliers in the dataset. (D) Elbow plot ranking variance explained by each principal component. (E) Spearman correlation between genes expressed between two replicates from the same treatment group. A correlation coefficient greater than 0.95 was found between replicates for all four treatments. (F) Uniform Manifold Approximation and Projection (UMAP) of nuclei from all the samples before batch correction. Nuclei from each replicated treatment are labeled with different colors. (G) UMAP of nuclei from the entire dataset after Harmony-based batch correction. After performing sample QC using Seurat [25, 26] and batch correction using Harmony [27], we obtained data from 56,000 nuclei across all samples with a median of 399 genes and 851 RNA molecules per nucleus.

Number of Genes per cluster

Total No. of RNA molecules per cluster

**Cluster#**

A

B

**Fig.S2.** **Metrics of single-nuclei sequencing data from *D. melanogaster* fat body.**

(A) Number of RNA molecules detected per cluster. Clusters (x-axis) represent 56,000 nuclei from eight samples. (B) Number of unique genes identified per cluster. The plot shows clusters (x-axis) from 56,000 nuclei sequenced across eight samples.

Cluster 0 - Yp3

Cluster 1 – Hsp27

Cluster 2 – Tsf1

Cluster 3 - Egfr

Cluster 4 - bt

Cluster 5 - mgl

Cluster 6 – FASN2

Cluster 7 - osk

Cluster 8 - Hml

Cluster 9 – me31B

Cluster 10 – Vm26Ab

Cluster 11 – Cp36

Cluster 12 – Mur18B

Cluster 13 - αTry

Cluster 14 – PPO2

Cluster 15 - para

Cluster 16 – CG16826

Cluster 17 – Cubn

Cluster 18 - Antp

**Fig.S3:** **UMAP of expression of cluster-specific markers**.


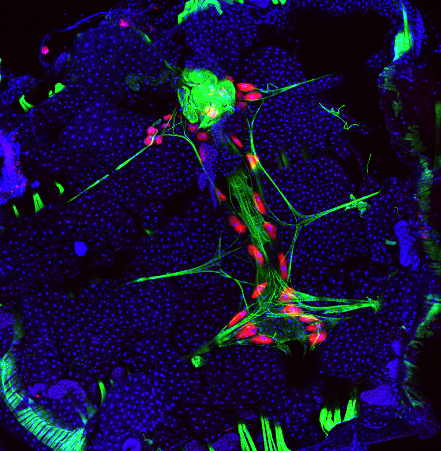


nACHR7α>mCherry

Fat Body

**Fig.S4.** **Spatial localization of Cluster 3**.

Expression of the fluorescent reporter mCherry under the control a nARCH*7a* driver labels putative Cluster 3 cells. The morphological and spatial profile of the cells expressing mCherry matched with that of pericardial cells [4, 5]. Nuclei are labelled with DAPI (blue) and actin is labeled with FITC-phalloidin (green).

Number of Clusters

Genes differentially expressed between VU-VI

B.

Proportion of differentially expressed genes

Number of Clusters

Genes differentially expressed between VU-MU

A.


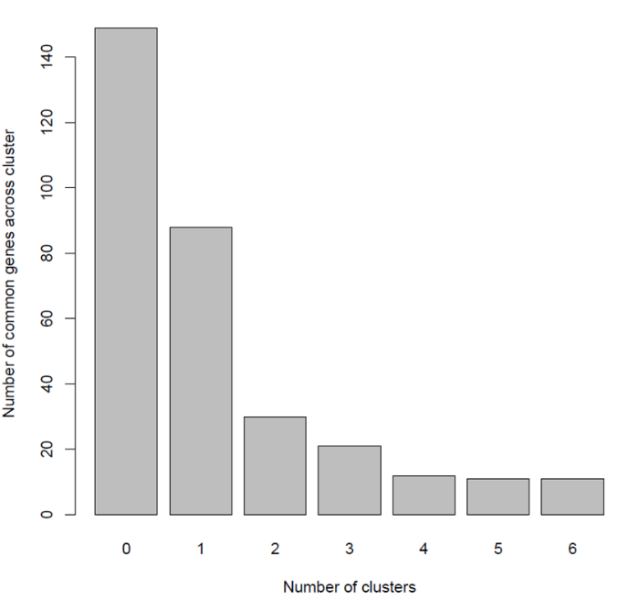


Number of differentially expressed genes

Genes differentially expressed between MU-MI

Number of Clusters

C.


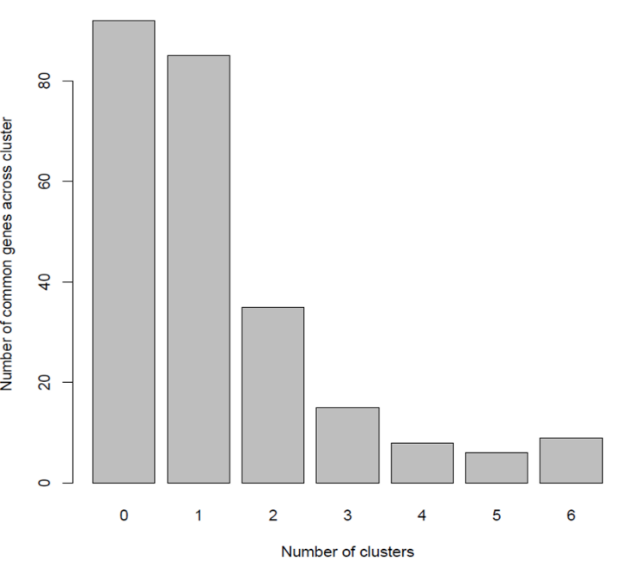
**Fig.S5.** **Frequency distribution of genes that are differentially expressed between treatments across six fat body clusters**.

(A) Proportion of differentially expressed genes shared between clusters upon mating (Virgin Uninfected (VU) vs Mated Uninfected (MU)). (B) Proportion of differentially expressed genes upon infection in virgin females (Virgin Uninfected (VU) vs Virgin Infected (VI)). (C) Proportion of differentially expressed genes upon infection in mated females (Mated Uninfected (MU) vs Mated Infected (MI)).

Number of differentially expressed genes

**Fig.S5D** **Heat map shows change in expression of genes with annotated immune function in response to infection in major fat body clusters (Clusters 0,1,2,5,7 and 10).**

VI / VU: Fold change in expression of genes in Virgin Infected relative to Virgin Uninfected

MI / MU: Fold change in expression of genes in Mated Infected relative to Mated Uninfected


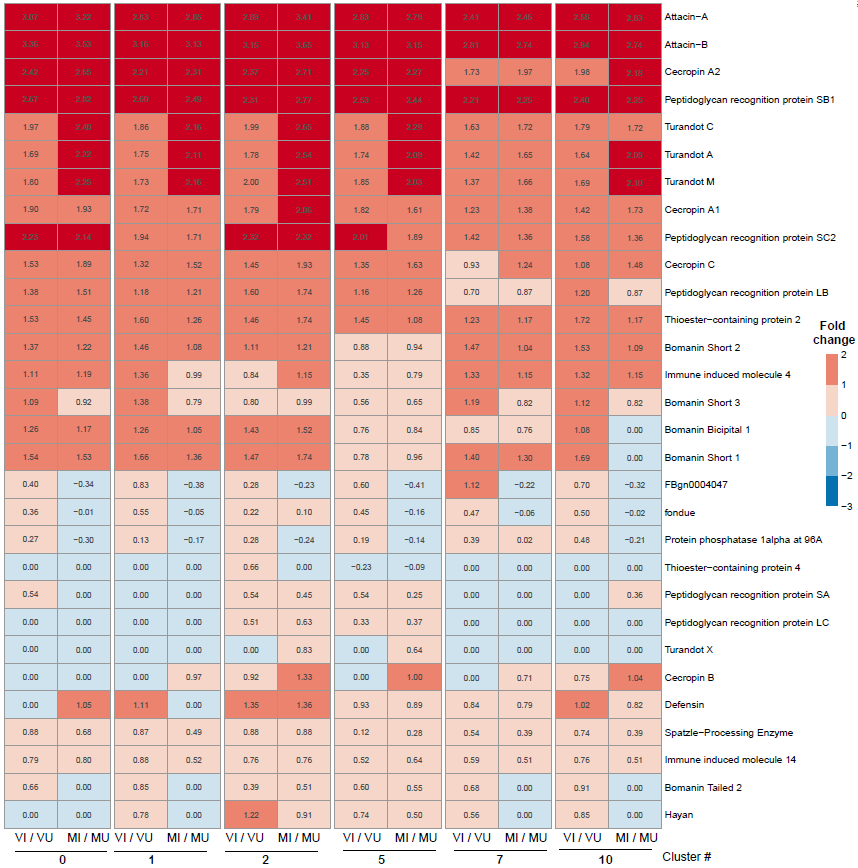


**
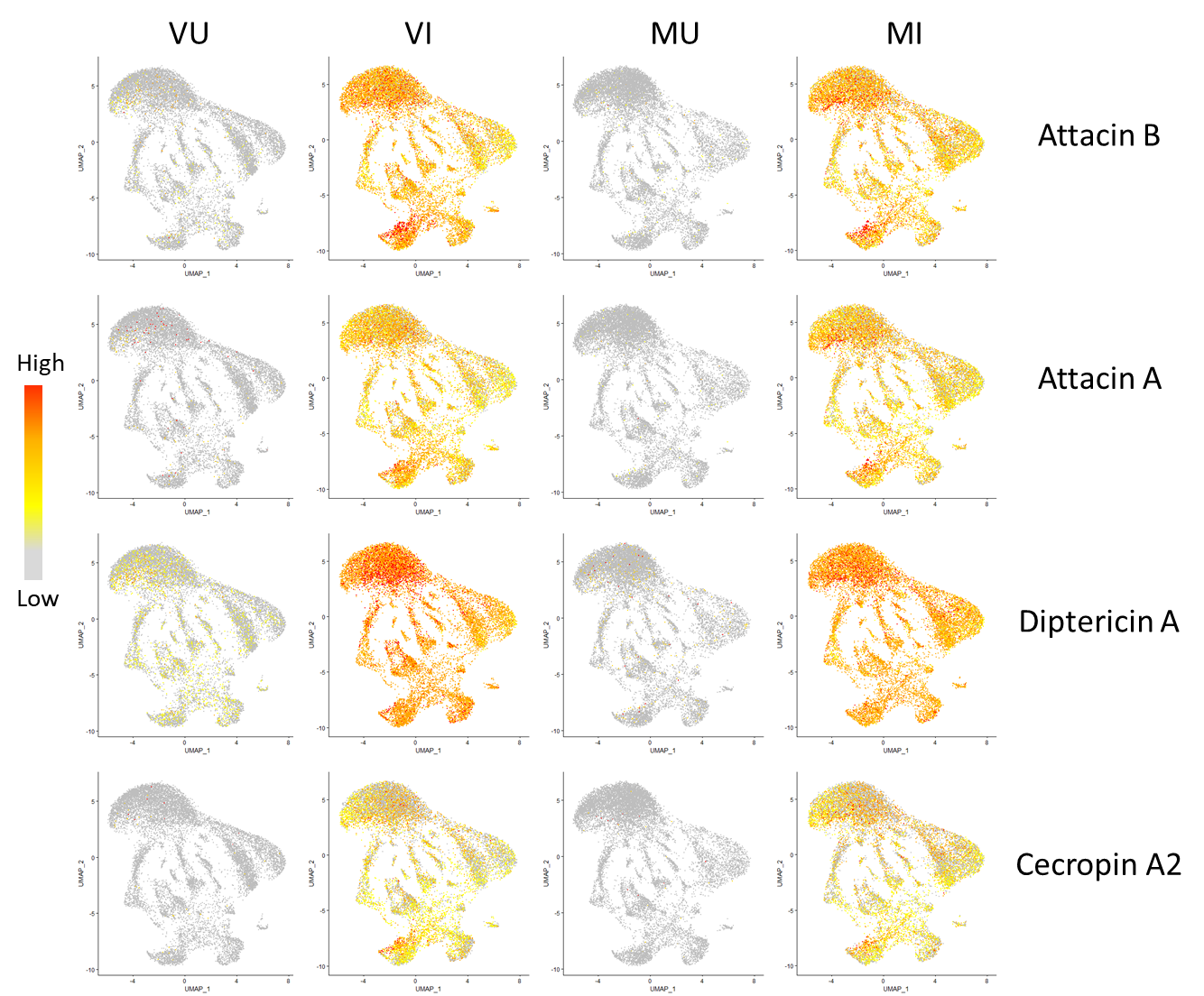
**

**Fig.S5E** **Feature plot show differences in gene expression of representative antimicrobial peptides across four treatments.**

Antimicrobial peptide gene expression is not restricted to a subset of fat body cells but is robustly induced over the entire fat body tissue and associated cells.


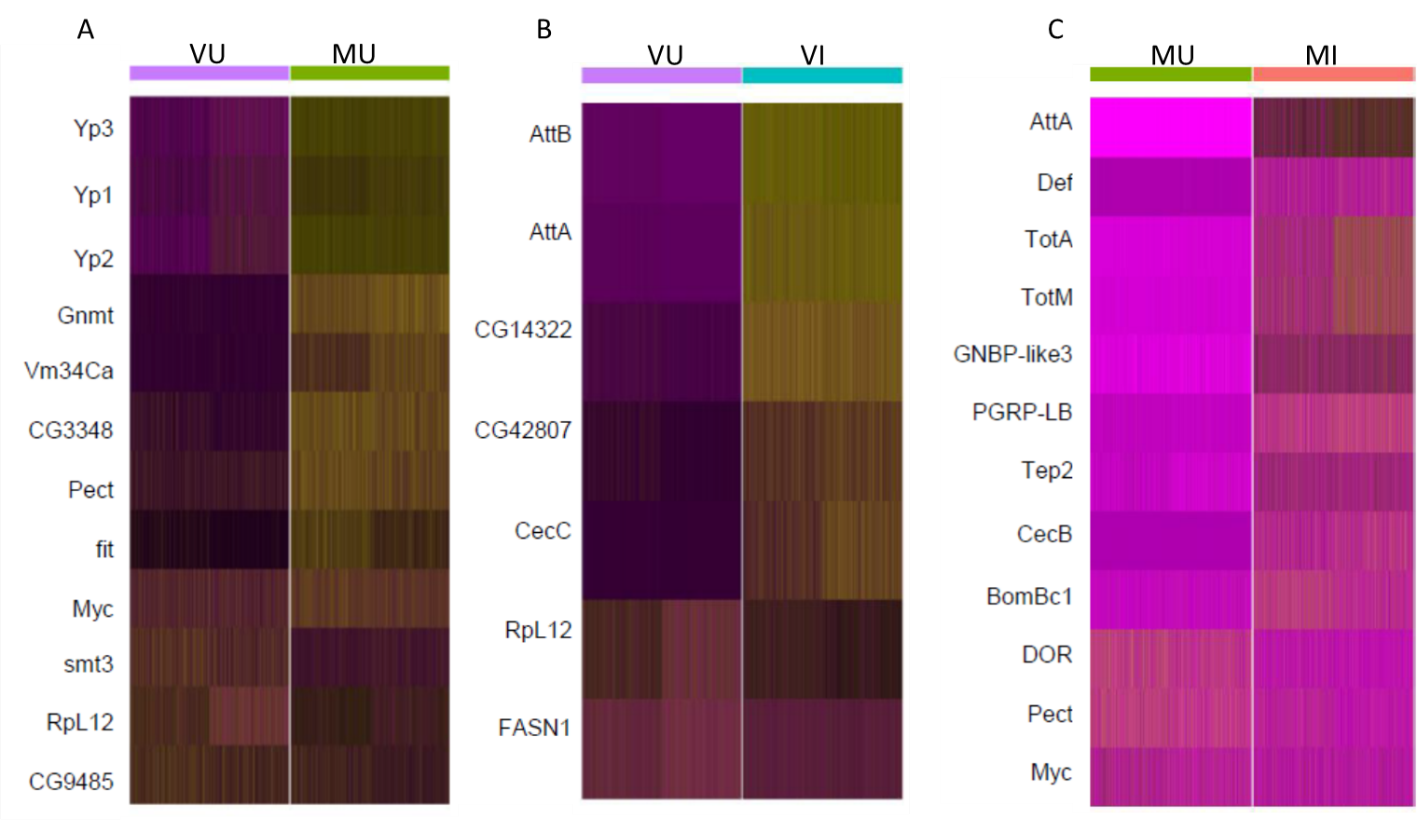


**Fig.S6.** **Heatmaps of top differentially expressed genes.**

(A) Upon mating (Virgin Uninfected (VU) vs Mated Uninfected (MU)), (B) Upon infection in Virgins (Virgin Uninfected (VU) vs Virgin Infected (VI)), and (C) Upon infection in Mated females (Mated Uninfected (MU) vs Mated Infected (MI)).

**Fig.S7.** **Trajectory analysis profiling of Drosophila fat body tissue**.

Monocle-based trajectory analysis separated nuclei into two disjointed trajectories (Trajectory 1 and Trajectory 2) primarily on the basis of their infection status. This indicates that nuclei from infected versus uninfected samples have dramatically different expression profiles. Different colors represent nuclei from four different treatments (Virgin Uninfected, Mated Uninfected, Virgin Infected, and Mated Infected).


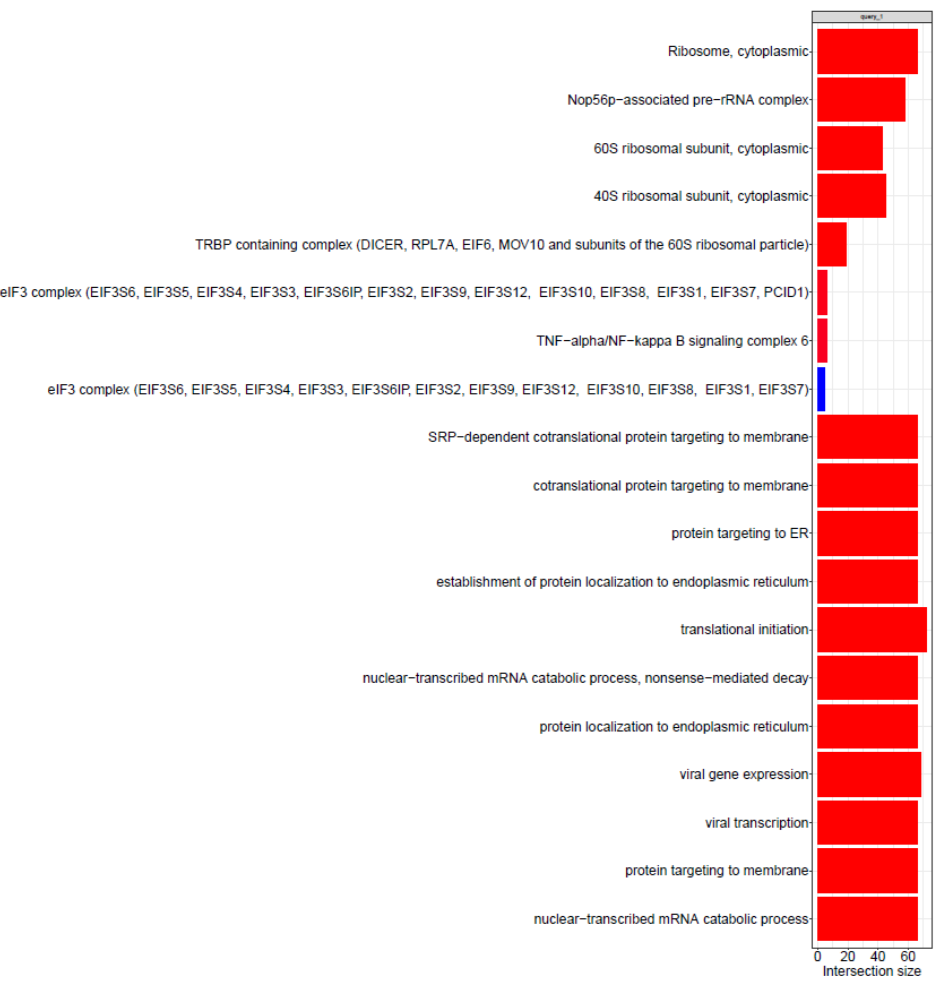


**Fig.S8.** **Enrichment plot of genes expressed in Module 13 obtained in pseudotime analysis**. Bar plot shows 20 most significantly enriched GO terms (p-adj). Enrichment was performed using g: profiler. P-adj: p-value for significant enrichment of GO terms after correction for multiple testing. Intersection size refers to the number of genes in the query that are annotated to each GO term. GO term analysis revealed enrichment for ribosome function and translation initiation in Module 13. Genes expressed in Module 13 showed lower gene expression score for Mated Uninfected compared to Virgin Uninfected (Fig. 2C).


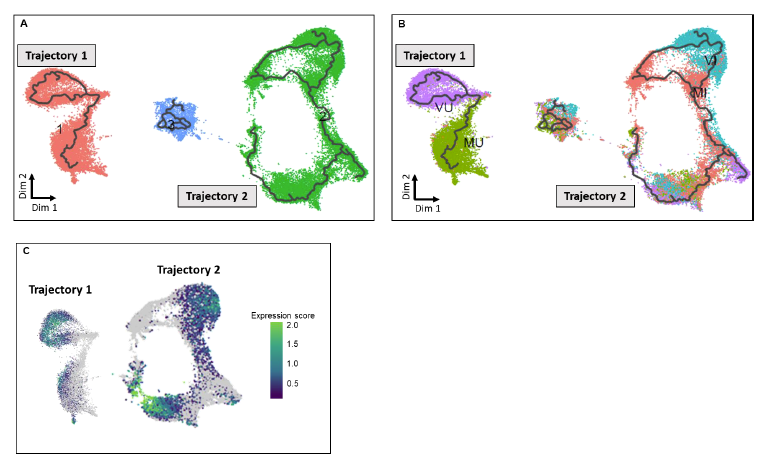


**D**

MI

VI

VU

MU

**Fig.S9.** **Trajectory analysis profiling a subset of clusters identified as fat body subpopulations (Clusters 0, 1,2,5,7 and 10)**.

(A) Monocle-based trajectory analysis on nuclei identified as fat body, separated nuclei into three disjointed trajectories. (B) Trajectory 1 and Trajectory 2 primarily separated on the basis of the infection status, consistent with the results shown in Fig.S7. (C) UMAP showing modules in each trajectory with lower gene expression aggregate scores for Mated Uninfected (Trajectory 1) and Mated Infected (Trajectory 2) compared to Virgin Uninfected and Virgin Infected respectively. (D) GO term analysis indicated enrichment for ribosome biogenesis and protein translation genes corroborating results shown in figure 2.

A.


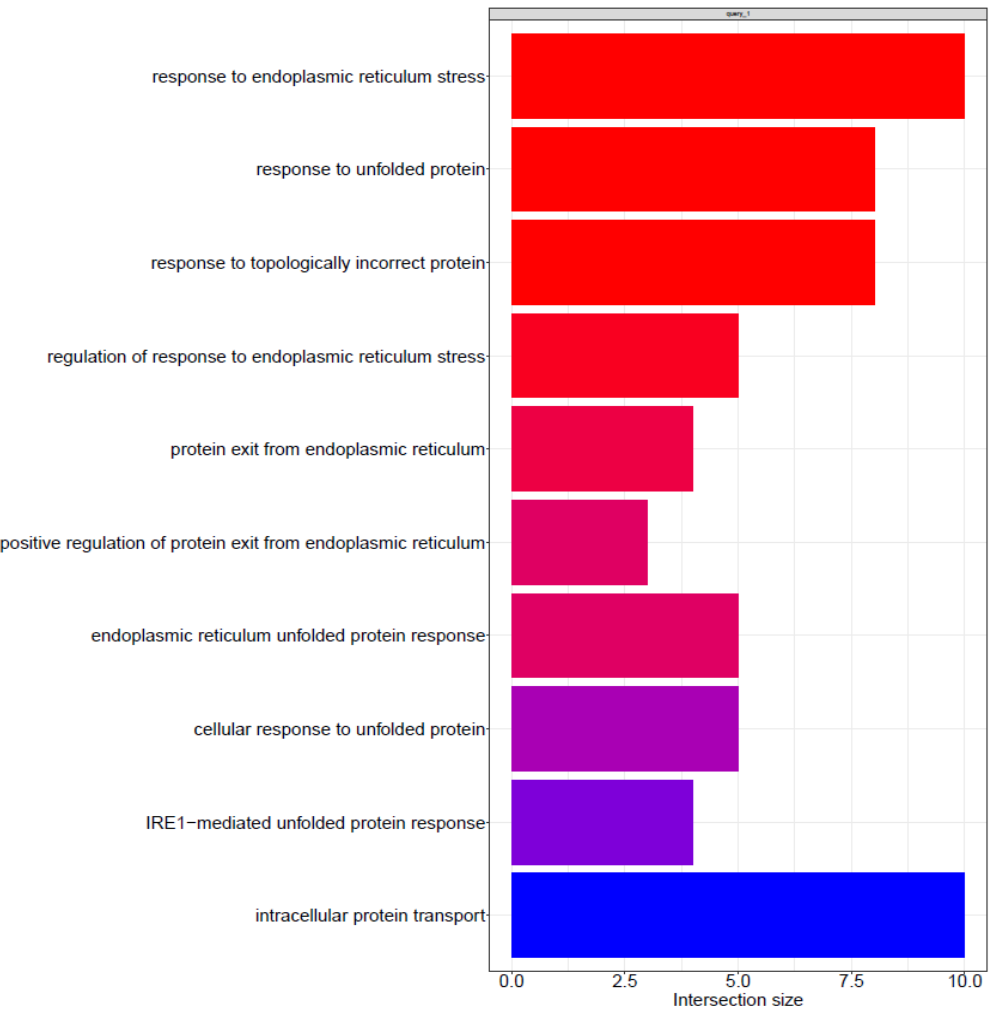


B.

**Fig.S10.** **Analysis of Module 15 (Trajectory 2) showing ER stress**.

(A) UMAP of Module 15 (Trajectory 2) showing high aggregate gene expression scores of genes present in Module 15 (Additional File 9) for a subset of Mated Infected (MI) nuclei. Gradient of color represents the aggregate expression score with light color indicating higher expression score. Each dot represents a single nucleus. (B) Bar plot shows 10 most significantly enriched GO terms (p-adj) for Module 15. P-adj: p-value for significant enrichment of GO terms after correction for multiple testing. Intersection size refers to the number of genes in the query that are annotated to each GO term. GO term analysis showed enrichment for endoplasmic reticulum stress in Module 15.


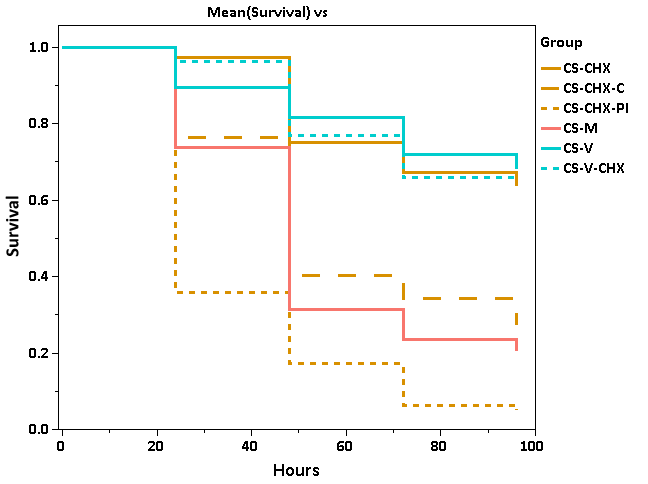


p < 0.0001


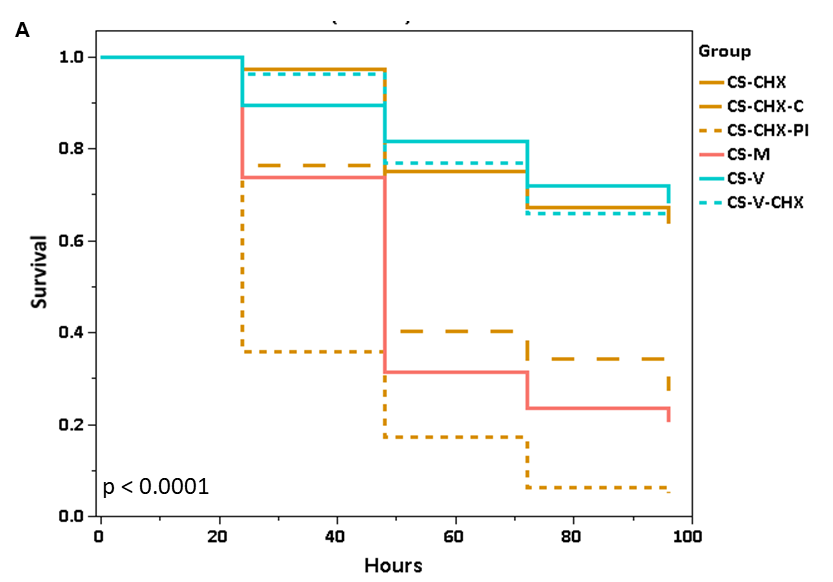


CHX

CHX - C

CHX - PI

MI

VI

CHX - VI

**Fig.S11.** **Survivorship analysis of post-infection mortality.**

Cox proportional hazard analysis showed that mated flies fed with Cycloheximide (CHX) **c**ontinuously (CHX-C) or only **p**ost-**i**nfection (CHX-PI) had significantly higher mortality compared to flies which were fed CHX only at the time of mating and before infection (CHX). Survivorship of virgin females fed with CHX (CHX-V) was not different from virgin females not fed CHX. Higher mortality of CHX-C and CHX-PI demonstrates that CHX does not provide any direct protection against infection with *Providencia rettgeri*. The CHX-C and CHX-PI treatments clearly demonstrate the necessity to synthesize proteins post-infection. (n = 35-40 flies per treatment per replicate, three replicates). p-value in the figure is the combined p-value of Cox proportional hazards analysis.

Table shows summary of results from risk ratios calculated between different treatments (Levels). Risk ratio is the ratio of hazard rates between two levels indicated as Level 1 and Level 2. Risk ratio < 1 shows lower risk to infection for level 1 and risk ratio > 1 shows higher risk to infection for level 1 – when compared to level 2.

**
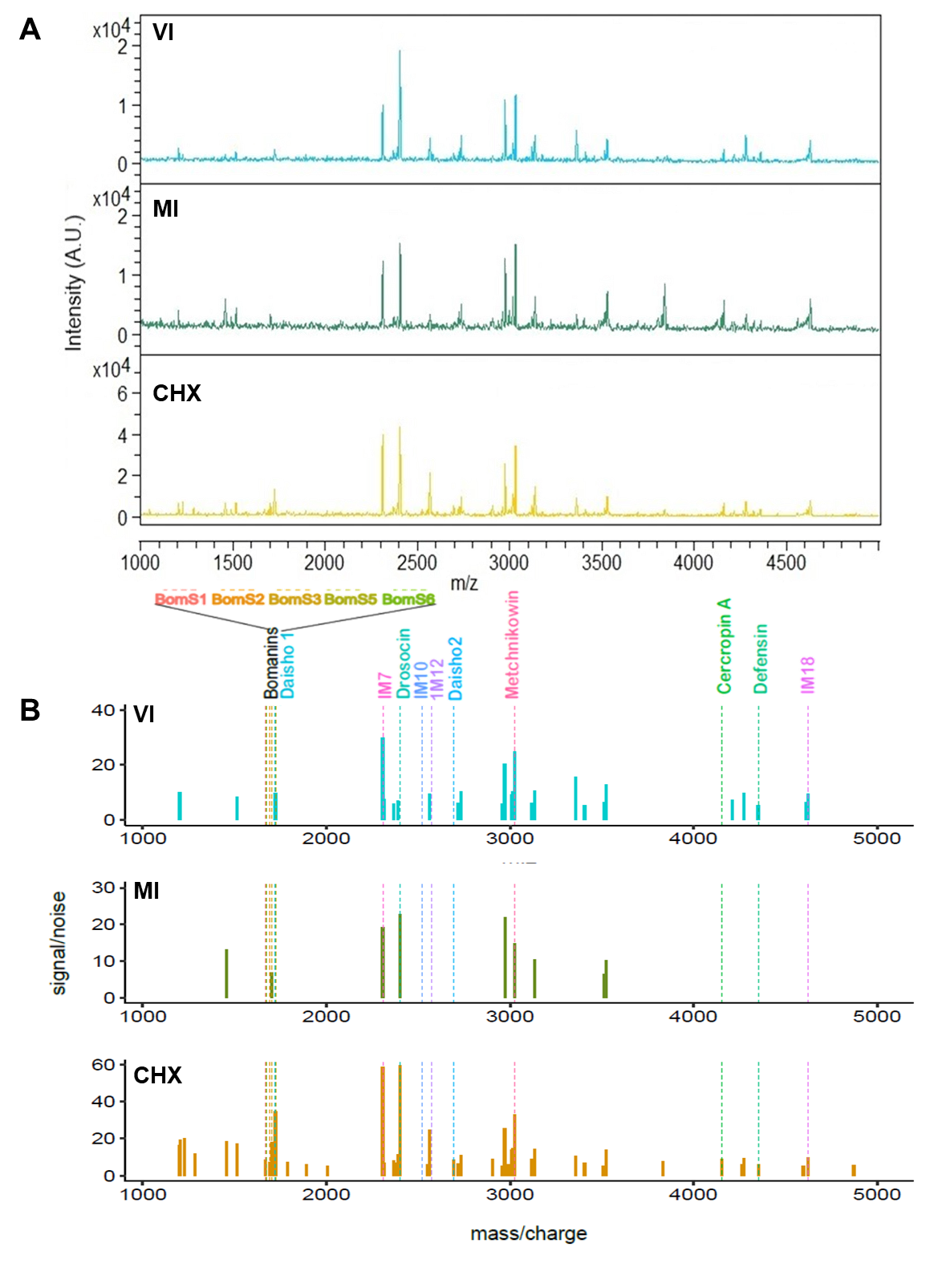
**

**Fig.S12. MALDI-TOF mass spectrometry-based peptide detection**

(A) Baseline subtracted and smoothened representative MALDI-TOF spectra between mass/charge (m/z) and intensity (A.U.) from VI, MI and CHX. (B) Plot between mass/charge and signal/noise ratio, used to detect peptides in the samples. Vertical lines indicate peptides with signal/ratio > 5 and dotted lines represent immune peptides detected across all the samples.

Table S1: (A) A summary of number of doublets identified in each sample. (B) Doublets were identified to be distributed across all clusters.

| Sample Name | Replicate# | Singles | Doublets |
| --- | --- | --- | --- |
| VU | 1 | 6564 | 436 |
| VU | 2 | 6562 | 438 |
| VI | 1 | 6552 | 448 |
| VI | 2 | 6574 | 426 |
| MU | 1 | 6559 | 441 |
| MU | 2 | 6565 | 435 |
| MI | 1 | 6549 | 451 |
| MI | 2 | 6550 | 450 |

| Cluster# | Singlets | Doublets |
| --- | --- | --- |
| 0 | 13554 | 616 |
| 1 | 6146 | 369 |
| 2 | 5677 | 276 |
| 3 | 5000 | 420 |
| 4 | 3559 | 306 |
| 5 | 3563 | 252 |
| 6 | 3328 | 355 |
| 7 | 3229 | 212 |
| 8 | 2348 | 211 |
| 9 | 1155 | 84 |
| 10 | 1134 | 76 |
| 11 | 977 | 83 |
| 12 | 758 | 92 |
| 13 | 651 | 59 |
| 14 | 344 | 37 |
| 15 | 309 | 19 |
| 16 | 299 | 16 |
| 17 | 232 | 27 |
| 18 | 212 | 15 |

Table S2: A summary of potential tissue type and markers representing each cluster. 19 clusters were identified from the entire dataset.

| **Cluster#** | **Potential tissue** | **Marker genes** |
| --- | --- | --- |
| 0 | Fat Body Cells | *yp3, yp1* |
| 1 | Fat Body Cells | *Hsp27, dhd* |
| 2 | Fat Body Cells | *CG31326, Nep6* |
| 3 | Unknown | *Pde9, Ubx* |
| 4 | Muscle Cells | *bt, sls* |
| 5 | Proliferative Fat Body Cells | *mgl, CG14661* |
| 6 | Oenocytes | *FASN2, CG7910* |
| 7 | Reproductive provisioning fat body cells | *osk,dhd* |
| 8 | Hemocytes | *Hml,Ten-m* |
| 9 | Uncharacterized | *me31B, Hsp27* |
| 10 | Structural fat body cells | *Vm26Ab, Vm26Aa* |
| 11 | Chorion producing fat body cells | *Cp36, Cp38* |
| 12 | Catabolic fat body cells | *Mur18b, CG14292* |
| 13 | Putative digestive cells | *alphaTry, Jon65Aiv* |
| 14 | Crystal cells | *PPO1, PPO2* |
| 15 | Neuronal | *Para, Rdl* |
| 16 | Stress response fat body cells | *whe, CG16826* |
| 17 | Nephrocytes | *Cubn, CG42255* |
| 18 | Tracheal cells | *Antp, trol* |

| **Cluster#** | **Potential tissue** | **Marker genes** |
| --- | --- | --- |
| 0 | Fat Body Cells | *yp3, yp1* |
| 1 | Fat Body Cells | *Hsp27, dhd* |
| 2 | Fat Body Cells | *CG31326, Nep6* |
| 3 | Unknown | *Pde9, Ubx* |
| 4 | Muscle Cells | *bt, sls* |
| 5 | Proliferative Fat Body Cells | *mgl, CG14661* |
| 6 | Oenocytes | *FASN2, CG7910* |
| 7 | Reproductive provisioning fat body cells | *osk,dhd* |
| 8 | Hemocytes | *Hml,Ten-m* |
| 9 | Uncharacterized | *me31B, Hsp27* |
| 10 | Structural fat body cells | *Vm26Ab, Vm26Aa* |
| 11 | Chorion producing fat body cells | *Cp36, Cp38* |
| 12 | Catabolic fat body cells | *Mur18b, CG14292* |
| 13 | Putative digestive cells | *alphaTry, Jon65Aiv* |
| 14 | Crystal cells | *PPO1, PPO2* |
| 15 | Neuronal | *Para, Rdl* |
| 16 | Stress response fat body cells | *whe, CG16826* |
| 17 | Nephrocytes | *Cubn, CG42255* |
| 18 | Tracheal cells | *Antp, trol* |

Table S3: A summary of the number of genes that are differentially expressed between treatments across 19 clusters. **VU-MU**: Number of genes differentially expressed upon mating (Virgin Uninfected (VU) vs Mated Uninfected (MU)), **VU-VI**: Number of genes differentially expressed upon infection in Virgins (Virgin Uninfected (VU) vs Virgin Infected (VI)), **MU-MI**: Number of genes differentially expressed upon infection in Mated (Mated Uninfected (MU) vs Mated Infected (MI)).

Clusters identified as fat body subpopulations are highlighted in grey color.

|  | Differentially expressed genes compared between | | | | | |
| --- | --- | --- | --- | --- | --- | --- |
|  | VU-MU | | VU-VI | | MU-MI | |
| Cluster# | Upregulated | Downregulated | Upregulated | Downregulated | Upregulated | Downregulated |
| 0 | 26 | 1 | 63 | 3 | 55 | 16 |
| 1 | 82 | 12 | 68 | 1 | 25 | 27 |
| 2 | 58 | 26 | 108 | 13 | 83 | 70 |
| 3 | 82 | 52 | 118 | 90 | 81 | 78 |
| 4 | 51 | 3 | 61 | 6 | 35 | 12 |
| 5 | 51 | 1 | 53 | 4 | 28 | 9 |
| 6 | 26 | 2 | 42 | 0 | 17 | 14 |
| 7 | 20 | 5 | 45 | 0 | 19 | 2 |
| 8 | 33 | 1 | 88 | 3 | 36 | 14 |
| 9 | 12 | 15 | 23 | 2 | 5 | 0 |
| 10 | 6 | 3 | 22 | 0 | 16 | 1 |
| 11 | 3 | 0 | 5 | 0 | 10 | 0 |
| 12 | 3 | 0 | 18 | 0 | 10 | 1 |
| 13 | 0 | 0 | 2 | 0 | 2 | 0 |
| 14 | 2 | 0 | 8 | 0 | 3 | 0 |
| 15 | 0 | 0 | 2 | 0 | 3 | 0 |
| 16 | 0 | 0 | 0 | 0 | 9 | 1 |
| 17 | 1 | 0 | 0 | 0 | 8 | 0 |
| 18 | 0 | 0 | 2 | 0 | 0 | 0 |

Table S4: (A) Effect of treatment (VU, VI, MU, and MI) on puromycin incorporation (relative fluorescence) measured using Western blotting. Summary of results of one-way ANOVA with Treatment using levels VU, VI, MU, and MI. Posthoc comparisons using Tukey’s HSD shows significantly different puromycin incorporation between VI and MI (Fig.4B). (B) Summary of results of pairwise contrast between VI and MI.

A.

| Source | DF | Sum of Squares | Mean Square | F Ratio | Prob > F |
| --- | --- | --- | --- | --- | --- |
| Treatment | 3 | 5.677495 | 1.8925 | 4.26 | 0.0216 |
| Error | 16 | 7.108 | 0.44425 |  |  |
| C. Total | 19 | 12.785495 |  |  |  |

B.

| Effect | SS | NumDF | DenDF | F Ratio | Prob > F |
| --- | --- | --- | --- | --- | --- |
| Genotype | 4.80249 | 1 | 16 | 10.8103 | 0.0046 |

Table S5: *t-test* results comparing effect of cycloheximide treatment on puromycin incorporation. VI represents Virgin Infected females, MI represents Mated Infected females not treated with cycloheximide and CHX represents females treated with CHX (See Methods).

| Treatment | | | | | |  |
| --- | --- | --- | --- | --- | --- | --- |
| MI | | | CHX | | | *t-test* |
| N | Mean | SD | N | Mean | SD | 3.6** |
| 5 | 1.02 | 0.60 | 5 | 1.59 | 0.88 |  |
| ***p* = 0.02 | |  |  |  |  |  |
|  | |  |  |  |  |  |

| Treatment | | | | | |  |
| --- | --- | --- | --- | --- | --- | --- |
| VI | | | MI | | | *t-test* |
| N | Mean | SD | N | Mean | SD | 5.9*** |
| 5 | 2.15 | 0.86 | 5 | 1.02 | 0.88 |  |
| ****p =* 0.004 | |  |  |  |  |  |

| Treatment | | | | | |  |
| --- | --- | --- | --- | --- | --- | --- |
| VI | | | CHX | | | *t-test* |
| N | Mean | SD | N | Mean | SD | 2.11 |
| 5 | 2.15 | 0.86 | 5 | 1.59 | 0.88 |  |
| *p* = 0.10 | |  |  |  |  |  |

Table S6: *t*-test results comparing number of peptides detected in (A) MI vs VI and (B) MI vs CHX treated flies.

| Treatment | | | | | |  |
| --- | --- | --- | --- | --- | --- | --- |
| MI | | | VI | | | *t-test* |
| N | Mean | SD | N | Mean | SD | 3.8** |
| 7 | 5 | 1.63 | 7 | 8.3 | 1.2 |  |
| ***p* = 0.009 | |  |  |  |  |  |

| Treatment | | | | | |  |
| --- | --- | --- | --- | --- | --- | --- |
| MI | | | CHX | | | *t-test* |
| N | Mean | SD | N | Mean | SD | 2.8** |
| 7 | 5 | 1.63 | 7 | 9.14 | 2.73 |  |
| ***p* = 0.03 | |  |  |  |  |  |

| Treatment | | | | | |  |
| --- | --- | --- | --- | --- | --- | --- |
| VI | | | CHX | | | *t-test* |
| N | Mean | SD | N | Mean | SD | -0.8 |
| 7 | 8.3 | 1.2 | 7 | 9.14 | 2.73 |  |
| *p* = 0.40 | |  |  |  |  |  |

**References**

1. Raudvere U, Kolberg L, Kuzmin I, Arak T, Adler P, Peterson H, et al. G:Profiler: A web server for functional enrichment analysis and conversions of gene lists (2019 update). Nucleic Acids Res. 2019;47:W191–8.

2. Hoy MA. Genetic Systems, Genome Evolution, and Genetic Control of Embryonic Development in Insects. 2019.

3. Jenett A, Rubin GM, Ngo TTB, Shepherd D, Murphy C, Dionne H, et al. A GAL4-Driver Line Resource for Drosophila Neurobiology. Cell Rep. 2012;2:991–1001. doi:10.1016/j.celrep.2012.09.011.

4. Hartley PS, Motamedchaboki K, Bodmer R, Ocorr K. SPARC-dependent cardiomyopathy in drosophila. Circ Cardiovasc Genet. 2016;9:119–29.

5. Rotstein B, Paululat A. On the Morphology of the Drosophila Heart. J Cardiovasc Dev Dis. 2016;3:15.

6. Christensen EI, Birn H. Megalin and cubilin: Multifunctional endocytic receptors. Nat Rev Mol Cell Biol. 2002;3:258–68.

7. Tsarouhas V, Yao L, Samakovlis C. Src kinases and ERK activate distinct responses to Stitcher receptor tyrosine kinase signaling during wound healing in Drosophila. J Cell Sci. 2014;127:1829–39.

8. Howick VM, Lazzaro BP. The genetic architecture of defence as resistance to and tolerance of bacterial infection in Drosophila melanogaster. Mol Ecol. 2017;26:1533–46.

9. Junell A, Uvell H, Davis MM, Edlundh-Rose E, Antonsson Å, Pick L, et al. The POU Transcription Factor Drifter/Ventral veinless Regulates Expression of Drosophila Immune Defense Genes. Mol Cell Biol. 2010;30:3672–84.

10. Halder G, Johnson RL. Hippo signaling: Growth control and beyond. Development. 2011;138:9–22.

11. Huang K, Chen W, Zhu F, Bai H. RiboTag translatomic profiling of Drosophila oenocytes under aging and oxidative stress. bioRxiv. 2018;:1–19.

12. Chen H, Zheng X, Zheng Y. Age-Associated Loss of Lamin-B Leads to Systemic Inflammation and Gut Hyperplasia. Cell. 2014;159:829–43. doi:10.1016/j.cell.2014.10.028.

13. Bosch PS, Makhijani K, Herboso L, Gold KS, Baginsky R, Woodcock KJ, et al. HHS Public Access surrounding tissues. 2020;51:787–803.

14. Dai J, Ma M, Feng Z, Pastor-Pareja JC. Inter-adipocyte Adhesion and Signaling by Collagen IV Intercellular Concentrations in Drosophila. Curr Biol. 2017;27:2729-2740.e4.

15. Dobson AJ, He X, Blanc E, Bolukbasi E, Yang M, Street G. Lifespan extension by dietary restriction in. bioRxiv. 2016.

16. Mauvezin C, Nagy P, Juhász G, Neufeld TP. Autophagosome-lysosome fusion is independent of V-ATPase-mediated acidification. Nat Commun. 2015;6 May.

17. Akam ME, Carlson JR. The detection of Jonah gene transcripts in Drosophila by in situ hybridization. EMBO J. 1985;4:155–61.

18. Yadav S, Eleftherianos I. Participation of the Serine Protease Jonah66Ci in the Drosophila Antinematode Immune Response. Infect Immun. 2019;87:1–17. doi:10.1128/IAI.00094-19.

19. Musselman LP, Fink JL, Ramachandran PV, Patterson BW, Okunade AL, Maier E, et al. Role of fat body lipogenesis in protection against the effects of caloric overload in drosophila. J Biol Chem. 2013;288:8028–42.

20. Bozler J, Kacsoh BZ, Chen H, Theurkauf WE, Weng Z, Bosco G. A systems level approach to temporal expression dynamics in Drosophila reveals clusters of long term memory genes. PLoS Genet. 2017;13:0–22.

21. Ghosh AC, Tattikota SG, Liu Y, Comjean A, Hu Y, Barrera V, et al. Drosophila pdgf/vegf signaling from muscles to hepatocyte-like cells protects against obesity. Elife. 2020;9:1–61.

22. Bras S, Martin-Lannerée S, Gobert V, Augé B, Breig O, Sanial M, et al. Myeloid leukemia factor is a conserved regulator of RUNX transcription factor activity involved in hematopoiesis. Proc Natl Acad Sci U S A. 2012;109:4986–91.

23. Senger K, Harris K, Levine M. GATA factors participate in tissue-specific immune responses in Drosophila larvae. Proc Natl Acad Sci U S A. 2006;103:15957–62.

24. Lee KZ, Lestradet M, Socha C, Schirmeier S, Schmitz A, Spenlé C, et al. Enterocyte Purge and Rapid Recovery Is a Resilience Reaction of the Gut Epithelium to Pore-Forming Toxin Attack. Cell Host Microbe. 2016;20:716–30.

25. Butler A, Hoffman P, Smibert P, Papalexi E, Satija R. Integrating single-cell transcriptomic data across different conditions, technologies, and species. Nat Biotechnol. 2018;36:411–20.

26. Stuart T, Butler A, Hoffman P, Hafemeister C, Papalexi E, Mauck WM, et al. Comprehensive Integration of Single-Cell Data. Cell. 2019;177:1888-1902.e21. doi:10.1016/j.cell.2019.05.031.

27. Korsunsky I, Millard N, Fan J, Slowikowski K, Zhang F, Wei K, et al. Fast, sensitive and accurate integration of single-cell data with Harmony. Nat Methods. 2019;16:1289–96. doi:10.1038/s41592-019-0619-0.
